# In vivo evidence for soluble Aβ oligomer-driven neuronal metabolic dysfunction in 5xFAD mice

**DOI:** 10.1186/s13195-026-02130-4

**Published:** 2026-07-11

**Authors:** Chaynita Dashora, Anant Bahadur Patel

**Affiliations:** 1 NMR Microimaging and Spectroscopy, CSIR-Centre for Cellular and Molecular Biology, Habsiguda, Uppal Road, Hyderabad, 500007 India; 2 Academy of Scientific and Innovative Research (AcSIR), Ghaziabad, 201002 India

**Keywords:** Alzheimer's disease, Amyloid-β oligomers, Astroglial metabolism, Gliosis, Neuronal metabolism, ^13^C NMR spectroscopy

## Abstract

**Background:**

Alzheimer’s disease (AD) is characterized by the extracellular accumulation of amyloid-β (Aβ). Brain glucose hypometabolism, an early feature of AD, reflects neuronal metabolic dysfunction before the onset of clinical symptoms. While Aβ plaques are central to AD pathogenesis, the mechanisms by which different Aβ species, such as plaques and soluble oligomers, drive cell-type-specific neurometabolic dysfunction during disease progression remain poorly understood. This study evaluated the roles of different Aβ species in the neurometabolic trajectory across the presymptomatic, symptomatic, and advanced stages of AD.

**Methods:**

5xFAD and wild-type (WT) mice were investigated for cognitive performance, quantitative Aβ levels, and glial activation markers at 3, 6, and 12 months of age. Neuronal and astroglial metabolic activities were evaluated ex vivo using state-of-the-art ^1^H-[^13^C]-NMR spectroscopy in conjunction with administration of [1,6-^13^C_2_]glucose and [2-^13^C]acetate, respectively.

**Results:**

The 5xFAD mice exhibited cognitive decline from 6 months that further deteriorated by 12 months. There was a marked increase in Aβ-plaque burden and soluble oligomers from 3 to 12 months of age. These mice showed a significant reduction in hippocampal glutamatergic (0.145 ± 0.031 vs. 0.172 ± 0.016 µmol/g/min, *p* = 0.035) and GABAergic neuronal metabolic activity (0.027 ± 0.006 v*s* 0.033 ± 0.003 µmol/g/min, *p* = 0.028) as early as 3 months of age, with similar reductions observed in the cerebral cortex. The neurometabolic impairments further aggravated with age. Astrocytic metabolic activity was not significantly changed till 6 months but was increased at 12 months in 5xFAD mice in both the cerebral cortex (0.101 ± 0.014 vs. 0.084 ± 0.006 µmol/g/min, *p* = 0.011) and hippocampus (0.103 ± 0.006 vs. 0.089 ± 0.012 µmol/g/min, *p* = 0.011). The increase in astrocytic metabolic activity paralleled reactive gliosis, suggesting a shift from early neuronal metabolic impairment to heightened astroglial metabolic activity and inflammatory glial responses during the advanced AD stage. Notably, Aβ_40_ oligomers exhibited a greater sensitivity to impairment in neuronal glucose oxidation and cognitive function than Aβ_42_ oligomers or fibrillar plaques.

**Conclusions:**

These findings delineate a sequential neurometabolic cascade during AD progression, characterized by early neuronal glucose hypometabolism, subsequent cognitive decline linked to soluble Aβ_40_ oligomers, and late-stage astroglial metabolic activation. Collectively, these results suggest that soluble Aβ_40_ oligomers exhibit the greatest sensitivity to neurometabolic and cognitive impairment during AD.

**Supplementary Information:**

The online version contains supplementary material available at https://doi.org/10.1186/s13195-026-02130-4.

## Background

Alzheimer’s disease (AD) accounts for approximately 60–70% of all dementia cases [1] and is marked by a gradual cognitive decline, accompanied by the accumulation of extracellular amyloid beta (Aβ) plaques and intracellular neurofibrillary tangles [2]. Increasing evidence points to soluble Aβ oligomers being the principal neurotoxic species capable of causing synaptic dysfunction and impairing long-term potentiation [3, 4] while driving cognitive decline in AD mouse models [5, 6]. Efficient brain function relies on tightly regulated oxidative energy metabolism to sustain synaptic transmission, neurotransmitter cycling, and synaptic activity [7, 8]. Disruption of cerebral glucose metabolism is a common feature across neurodegenerative disorders [9], yet the cellular origin, temporal onset, and progression of oxidative metabolic failure across different brain cell types remain incompletely understood.

^18^F-fluoro-deoxyglucose (FDG) positron emission tomography (PET) studies have consistently demonstrated glucose hypometabolism in AD-vulnerable brain regions [10, 11]. However, this approach provides a macroscopic view of cerebral glucose consumption but lacks the capability to assess mitochondrial function and energy metabolism at the cellular level. Recent advances in multinuclear NMR spectroscopic approaches, combined with the administration of stable-isotope-labeled substrates, have enabled precise, cell-specific metabolic analysis in the brain [12–14]. These studies revealed reduced neuronal glucose metabolism, altered glial metabolic activity, and reduced neurotransmitter cycling between neurons and astrocytes in AD [12, 15].

5xFAD mouse model recapitulates key features of human AD, including early cognitive decline, neuroinflammation, synaptic dysfunction, and neuronal loss [16, 17]. Nevertheless, neurometabolic investigations in 5xFAD mice using mass spectrometry-based ^13^C flux analyses [18] and PET imaging [19, 20] reported inconsistent results, reflecting differences in analytical resolution and disease staging. These inconsistencies highlight the need for longitudinal, cell-type-specific analyses to delineate how amyloid pathology alters neuronal and astroglial energy metabolism across disease progression.

To address this gap, a stage-resolved analysis was performed to quantify Aβ plaques, soluble Aβ oligomers, neuron- and astrocyte-specific metabolic fluxes, cognitive function, and neuroinflammatory markers across the presymptomatic, symptomatic, and advanced stages of AD in 5xFAD mice. The neuronal and astroglial metabolic fluxes were measured in the cerebral cortex and hippocampus using ^1^H-[^13^C]-NMR spectroscopy with cell-type specificity. This integrated approach aimed to define how cerebral energy metabolism is reorganized in a cell-specific manner during AD progression and how distinct pathological features, including different Aβ species, gliosis, and cognitive decline, relate to these metabolic alterations. The findings indicate early impairment in neuronal glucose oxidation, whereas astrocytic oxidative metabolism is enhanced at the advanced stage.

## Methods

### Animals

All mice were maintained under a 12–12 h light-dark cycle with *ad libitum* access to food and water. All the experimental procedures with mice were approved by the Institutional Animals Ethics Committee (Protocol Nos. IAEC#41/2024, 27/2023, 66/2022) of the CSIR-Centre for Cellular and Molecular Biology, Hyderabad, India, and were conducted per the guidelines established by the Committee for Control and Supervision on Experiments on Animals, Ministry of Fisheries and Dairying, Government of India. 5xFAD mice of B6SL background (MMRRC Strain # 034848-JAX, Jackson Laboratory) were used in the study. The mice were bred by crossing hemizygotes to wild-type (WT) littermates. To avoid complications arising from female hormonal fluctuations on experimental measures, only male mice were used in the study. In total 117 mice were studied at 3 months (5xFAD, *n* = 20; WT, *n* = 20), 6 months (5xFAD, *n* = 20; WT, *n* = 19), and 12 months (5xFAD, *n* = 19; WT, *n* = 19). No blinding was involved as all the experimental procedures and data analysis were carried out by the same individual(s). ARRIVE guidelines were followed in the preparation of the manuscript.

### Assessment of cognitive function

The learning and memory in the individual cohort of mice were assessed at 3 months (5xFAD, *n* = 8; WT, *n* = 8), 6 months (5xFAD, *n* = 15; WT, *n* = 16), and 12 months (5xFAD, *n* = 15; WT, *n* = 15) of age. Animal movement was tracked and analyzed using Noldus EthoVision XT in the following behavioral tests:

#### Morris water maze test

Learning and memory in mice were evaluated using the Morris water maze (MWM) test [21]. The maze consisted of a circular tank (diameter: 150 cm, height: 60 cm), divided into four quadrants, each marked with distinct visual cues on the surrounding wall. The pool was filled to three-fourths of its volume with water, and a hidden escape platform was submerged one cm below the water in one of the quadrants. Mice were trained for four consecutive days, with three sessions per day (90 s each), starting from different quadrants to locate the hidden platform. After a 2-day interval, memory retention was assessed on the seventh day by measuring the escape latency of animals.

##### Y-maze test

Spatial memory was assessed using the Y-maze test [22]. Briefly, each mouse was placed in the center of a Y-shaped maze and allowed to explore for 8 min. The sequence of arm entries was recorded, and the spontaneous alternation (SA) was calculated as follows:

1 $$\:Spontaneous\:\:Alternation\:\left(\%\right)=\:\frac{Number\:of\:alternating\:triads}{Total\:number\:of\:triads}\times100$$

### Histology and immunostaining

A separate group of mice was used to analyze Aβ plaque burden and glial pathology at 3, 6, and 12 months of age, with 4 mice per genotype at each age. Mice were anesthetized with a ketamine:xylazine mixture (100:16 mg/kg, intraperitoneally), and transcardially perfused with ice-cold phosphate-buffered saline (PBS). One hemibrain was post-fixed by incubation in 4% paraformaldehyde (PFA) in PBS, whereas the other hemisphere was used to measure Aβ oligomers. For immunostaining, brains were subsequently cryoprotected in 30% glycerol. Coronal sections (30 μm) obtained using a cryotome (Leica Biosystems) were mounted on charged slides, boiled in citrate buffer (pH 6.0) for 20 min for antigen retrieval, blocked with 3% normal goat serum at room temperature (RT) for 2 h, and then processed for immunostaining.

**Immunohistochemistry:** The slides were incubated overnight at 4 °C with an anti-Aβ plaque antibody (1:250; Abcam, ab201060), followed by biotinylated secondary antibody (1:250; PK-6101) for 2 h at RT. Brain sections were further incubated with Avidin-Biotin complex (VectorLabs, PK-6101) for 2 h at RT, followed by staining using 3,3’-diaminobenzidine (VectorLabs, PK-6105). The stained sections were visualized using a Zeiss stereo microscope at 14x magnification. Aβ plaque load and burden in the cerebral cortex and hippocampus were quantified using ImageJ software [23].

**Immunofluorescence:** The brain sections were incubated overnight at 4 °C with anti-glial fibrillary acidic protein (GFAP) conjugated with FITC (1:200; Invitrogen, MA545632) and anti-IBA1 (1:200; Cell Signaling Technology, 17198), followed by donkey anti-rabbit secondary antibody conjugated with Alexa 555 (1:200; Invitrogen, A31572), for astrocytes and microglia, respectively. The slides were sealed with Fluoroshield mounting medium containing diamidinophenylindole (DAPI; Abcam, ab104139). The CA1, CA3, DG, and cerebral cortex regions were imaged using a confocal microscope (Leica TCS SP8 STED) equipped with a 63x oil-immersion objective. Images were processed using the LasX software (Leica Microsystems). The number and area of cells were counted using ImageJ.

### Quantification of Aβ_40_ and Aβ_42_ in brain homogenates

Total protein was isolated from brain tissues as described previously [24]. Briefly, brain tissue was homogenized with a tissue grinder in 800 µL buffer (pH 7.8) containing Tris-HCl (20 mM), ethylenediaminetetraacetic acid (5 mM), and a protease inhibitor cocktail (PIC; Roche Diagnostics, Mannheim, Germany). The homogenate was centrifuged at 16,000 g for 30 min at 4 °C, and the supernatant was further centrifuged at 1,00,000 g for 1 h. The resulting pellet was dissolved in 5 M Guanidine-HCl (GuHCl), incubated for 4 h at 30 °C on a shaker, and centrifuged at 1,00,000 g for 1 h at 4 °C. The total protein in the Tris-soluble and GuHCl-soluble fractions was determined using the Micro BCA^™^ Protein Assay kit (Pierce Biotechnology, Rockford, IL, USA). Levels of Human Aβ_40_ and Aβ_42_ oligomers were measured using enzyme-linked immunosorbent assay (ELISA) kits (E-EL-H0542, E-EL-H0543).

### Infusion of [1,6-^13^C_2_]glucose and [2-^13^C]acetate

Male 5xFAD and WT mice that had undergone cognitive assessments were used for neurometabolic measurements. Neuronal metabolic analysis was carried out by infusing [1,6-^13^C_2_]glucose at 3 months (5xFAD, *n* = 8; WT, *n* = 8), 6 months (5xFAD, *n* = 8; WT, *n* = 8), and 12 months (5xFAD, *n* = 7; WT, *n* = 7). The astroglial metabolic analysis was performed by [2-^13^C]acetate administration at 3 months (5xFAD, *n* = 8; WT, *n* = 8), 6 months (5xFAD, *n* = 7; WT, *n* = 8) and 12 months (5xFAD, *n* = 8; WT, *n* = 8). For these measurements, mice fasted for 5 h were anesthetized with urethane (1.5 g/kg, intraperitoneally). The body temperature (~ 37 °C) of animals was maintained using a heating pad connected to a temperature-regulated circulating water bath. The ^13^C-labeled substrates were infused via the tail vein using a programmable infusion pump (Harvard Apparatus, MA, USA). In brief, [1,6-^13^C_2_]glucose (0.225 mol/L) or [2-^13^C]acetate (1 mol/L, Cambridge Isotope Laboratories, Andover, MA, USA) was intravenously administered as a bolus variable infusion for 10–15 min, respectively [25]. Plasma was collected from blood withdrawn from the retro-orbital sinus during the last minute of infusion. The brain metabolism was arrested at the end of the infusions using a Focused Beam Microwave Irradiation system at 3.0 kW for 1.2 s (MMW-05, Muromachi Kikai Co Ltd., Japan). Brain regions including the cerebral cortex and hippocampus were dissected out, snap-frozen in liquid nitrogen, and stored at -80 °C until further analysis.

### NMR analysis of blood plasma

Plasma (100 µL) was mixed with sodium phosphate buffer (450 µL) prepared in D_2_O containing 1 mmol/L formate, and the mixture was filtered using a 10 kDa cutoff centrifugal filter to remove macromolecules. The glucose and acetate concentrations were measured in the ^1^H NMR spectrum of plasma obtained at 600 MHz NMR spectrometer (Bruker Biospin, Germany), using formate as an internal reference. The percent ^13^C labeling of glucose-C1α (5.2 ppm) and acetate-C2 (1.9 ppm) was calculated as the ^13^C signal intensity divided by the total (^12^C+^13^C) signal intensity above natural abundance.

### NMR analysis of brain metabolites

The brain metabolites were extracted from the cortex and hippocampus using the ethanol extraction protocol [26]. Briefly, brain tissue was weighed and homogenized with 0.1 N HCl in methanol (2:1 vol: wt) using a battery-operated homogenizer. [2-^13^C]Glycine (100 µL, 2 mmol/L) was added as an internal concentration reference and further homogenized with 90% ethanol in phosphate buffer (6x) on ice. The homogenate was centrifuged at 15,000 g for 45 min at 4°C. The supernatant was lyophilized and dissolved in 550 µL D_2_O containing 0.25 mmol/L sodium 3-(trimethylsilyl)-2,2’,3,3’-D4-propionate (TSP) for NMR analysis. The concentrations and ^13^C enrichments of metabolites were measured in ^1^H-[^13^C]-NMR spectra of brain tissue extracts recorded on a 600 MHz NMR spectrometer [27]. The cerebral metabolite levels were determined from the area under the respective peak in the unedited spectrum (^1^H-[^12^C+^13^C]) relative to [2-^13^C]glycine in the ^13^C-edited spectrum. The percentage ^13^C labeling of metabolites was calculated as the ratio of the intensity in the ^13^C-edited spectrum to that in the unedited spectrum.

### Determination of glucose oxidation rate

[1,6-^13^C_2_]Glucose is metabolized in neurons (glutamatergic and GABAergic) and astroglia to pyruvate-C3 (Pyr_C3_) by glycolysis and enters the respective TCA cycles *via* acetyl-CoA-C2 (AcCoA_c2_) [25, 28]. The ^13^C-label is transferred through TCA cycle intermediates to α-ketoglutarate-C4 (α-KG_C4_), which subsequently undergoes rapid transamination via glutamate dehydrogenase to form glutamate-C4 (Glu_C4_). Subsequent metabolism of α-KG_C4_ (Glu_C4_) through the TCA cycle results in equal labeling of oxaloacetate-C2 (OAA_C2_) and oxaloacetate-C3 (OAA_C3_), which ultimately transfer the label to Glu_C2_ and Glu_C3_ equally during the subsequent turn of the TCA cycle. Asp_C2_ and Asp_C3_ are labeled through rapid exchange with OAA_C2_ and OAA_C3_, respectively. The ^13^C labeled glutamate released from glutamatergic neurons is transported into astroglia and converted to glutamine by glutamine synthetase, thereby labeling glutamine at the corresponding positions (Gln_C4_/Gln_C3_/Gln_C2_) through glutamate-glutamine cycling. In GABAergic neurons, Glu_C4_ is decarboxylated to GABA_C2_, which subsequently contributes to Gln_c4_ labeling *via* GABA-Gln cycling. GABA_C3_ and GABA_C4_ are labeled following subsequent metabolism of α-KG_C4_ (Glu_C4_/GABA_C2_) by GABAergic TCA cycle. The rate of glucose oxidation in glutamatergic neurons (CMR_Glc(Glu)_) was estimated from ^13^C trapped into amino acids from [1,6-^13^C_2_]glucose over 10 min [29, 30]:

2 $$\begin{aligned}&\:{CMR}_{Glc\left(Glu\right)}=\:0.5\times\:\left(\frac{1}{10}\right)\\&\times\:\left\{0.82\times\:\left({Glu}_{\mathrm{C}4}+{2\times\:Glu}_{\mathrm{C}3})+0.42\times\:\left(2\times\:{Asp}_{\mathrm{C}3}\right)\}\:\right.\right.\end{aligned}$$

Glu_C4_, Glu_C3_, and Asp_C3_ represent concentrations of ^13^C-labeled glutamate and aspartate at the respective carbon positions for 100% [1,6-^13^C_2_]glucose in blood plasma. The fractions 0.82 and 0.42 represent glutamate and aspartate pools, respectively, in glutamatergic neurons [25].

Similarly, the rate of glucose oxidation in GABAergic neurons (CMR_Glc(GABA)_) was determined as:

3 $$\begin{aligned}&\:{CMR}_{Glc\left(GABA\right)}=\:0.5\times\:\left(\frac{1}{10}\right)\\&\times\:\left\{0.02\times\:\left({Glu}_{\mathrm{C}4}+{2\times\:Glu}_{\mathrm{C}3})+\mathrm{G}{ABA}_{\mathrm{C}2}+2\:{\times\:GABA}_{\mathrm{C}4}+0.42\times\:\left(2\times\:{Asp}_{\mathrm{C}3}\right)\}\:\right.\right.\end{aligned}$$

Asp_Ci_, GABA_Ci_, and Glu_Ci_ represent the ^13^C concentrations of aspartate, GABA, and glutamate, respectively at the i^th^ carbon positions.

### Determination of acetate oxidation rate

Acetate is preferentially transported and metabolized by astrocytes [31]. Thus, ^13^C-labeled acetate has been routinely used to assess the astroglial metabolic activity in rats [32] and humans [33]. Oxidation of [2-^13^C]acetate in astroglial TCA cycle incorporates label into α-KG_C4_, which transfers ^13^C into a small pool of Glu through rapid exchange, thus labeling Glu_C4_. Gln_C4_ is labeled by the activity of glutamine synthetase. GABA_C2_ and larger Glu_C4_ pool are subsequently labeled through GABA-glutamine and glutamate-glutamine cycles, respectively [32]. The cerebral metabolic rate of acetate oxidation (CMR_Ace(Ox)_) was calculated from the ^13^C trapped into amino acids in 15 min of [2-^13^C]acetate infusion [29]:

4 $$\begin{aligned}&\:{CMR}_{Ace\left(Ox\right)}=\:\left(\frac{1}{15}\right)\\&\:\times\:\left\{{Glu}_{\mathrm{C}4}+{GABA}_{\mathrm{C}2}+{Gln}_{\mathrm{C}4}+\:2\left({{Asp}_{\mathrm{C}3}+\:Glu}_{\mathrm{C}3}+{GABA}_{\mathrm{C}4}\right)\right\}\end{aligned}$$

Asp_i_, GABA_i_, Gln_i_, and Glu_i_ are the concentrations of ^13^C-labeled amino acids at the ‘i^th^’ carbon position for 100% [2-^13^C]acetate in blood plasma.

### Statistical analysis

Statistical analyses were carried out using GraphPad Prism *v8.0*. Normality of data was established using the Kolmogorov-Smirnov test. Statistical significance of different measures between 5xFAD and WT mice was determined using Student’s *t*-test with appropriate corrections for multiple comparisons. For data that did not follow a normal distribution, the Mann-Whitney U test with appropriate corrections for multiple-comparison was used. Data are presented as mean±standard deviation (SD). A p-value < 0.05 was considered statistically significant.

## Results

### Progressive cognitive impairment in 5xFAD mice

**Fig. 1:**
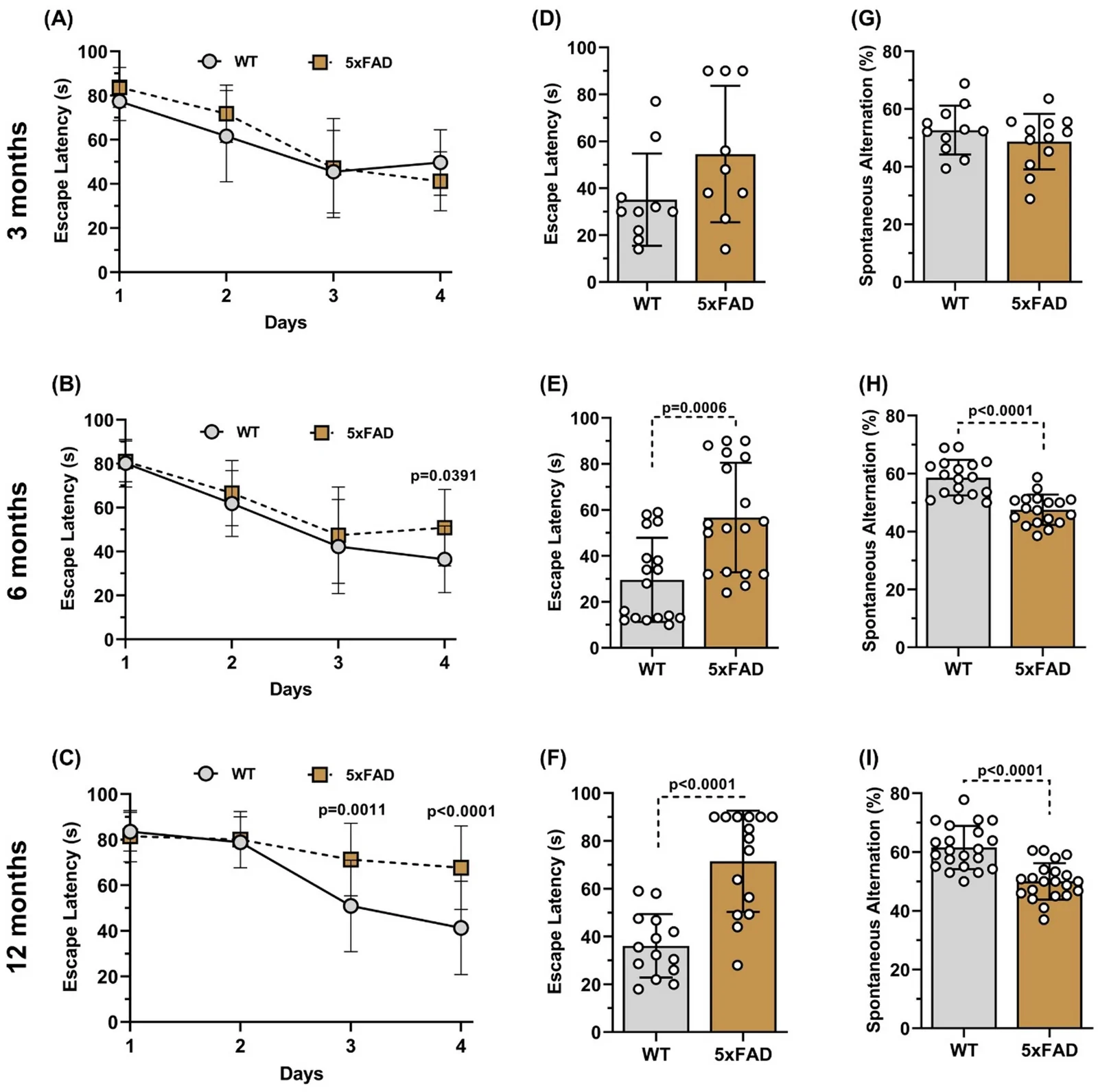
Learning and memory in 5xFAD and WT mice at different ages. **A-C** Learning performance of 5xFAD (*n* = 9–18) and WT (*n* = 10–17) mice at different ages in the Morris water maze test. **D-F** Memory in 5xFAD mice. **G-I** Working memory of 5xFAD and WT mice assessed using Y-Maze test. Each data point represents a measurement from an individual animal. Data are shown as mean ± SD. Statistical significance between groups was determined using one-way ANOVA for learning and unpaired Student’s T-test for memory tests

To evaluate stage-dependent alterations in learning and memory, 3-, 6-, and 12-month-old 5xFAD and wild-type (WT) mice were subjected to MWM and Y-maze tests. During the MWM training, escape latency of 3-month-old 5xFAD mice (44.3 ± 10.4 s, *n* = 8) was comparable to WT mice (53.4 ± 14.4 s, *n* = 8; *p* = 0.649; Fig. 1A). However, by 6 months, 5xFAD mice displayed significantly prolonged escape latencies (52.1 ± 17.5 s, *n* = 15) relative to WT (36.5 ± 15.7 s, *n* = 16; *p* = 0.0394), and this deficit was further exacerbated at 12 months (68.4 ± 20.1 s, *n* = 15 vs. 41.3 ± 20.5 s, *n* = 15; *p* < 0.001; Fig. 1B, C).

In the memory test, at 3-months of age, there was no significant (*p* = 0.351) difference in the escape latency of 5xFAD mice (50.1 ± 27.7 s) compared to WT mice (39.9 ± 19.1 s; Fig. 1D). By 6 months, memory declined markedly, with 5xFAD mice showing significantly higher escape latency than WT mice (55.6 ± 22.1 s vs. 29.5 ± 18.3 s, *p* < 0.001; Fig. 1E). This deficit further worsened at 12 months (66.7 ± 25.0 s vs. 38.0 ± 17.9 s, *p* < 0.001; Fig. 1F). Consistent changes were observed in the Y-maze spontaneous alternation, where 5xFAD mice displayed intact spatial memory at 3 months (*p* = 0.306; Fig. 1G) but pronounced impairments at 6 months (*p* < 0.001) and 12 months (*p* < 0.001; Fig. 1H, I).

Together, these behavioral data delineate a clear temporal pattern of cognitive decline in 5xFAD mice, with 3, 6, and 12 months corresponding to presymptomatic, symptomatic, and advanced AD stages, respectively.

### Age-dependent accumulation of Aβ species in 5xFAD mice

**Fig. 2:**
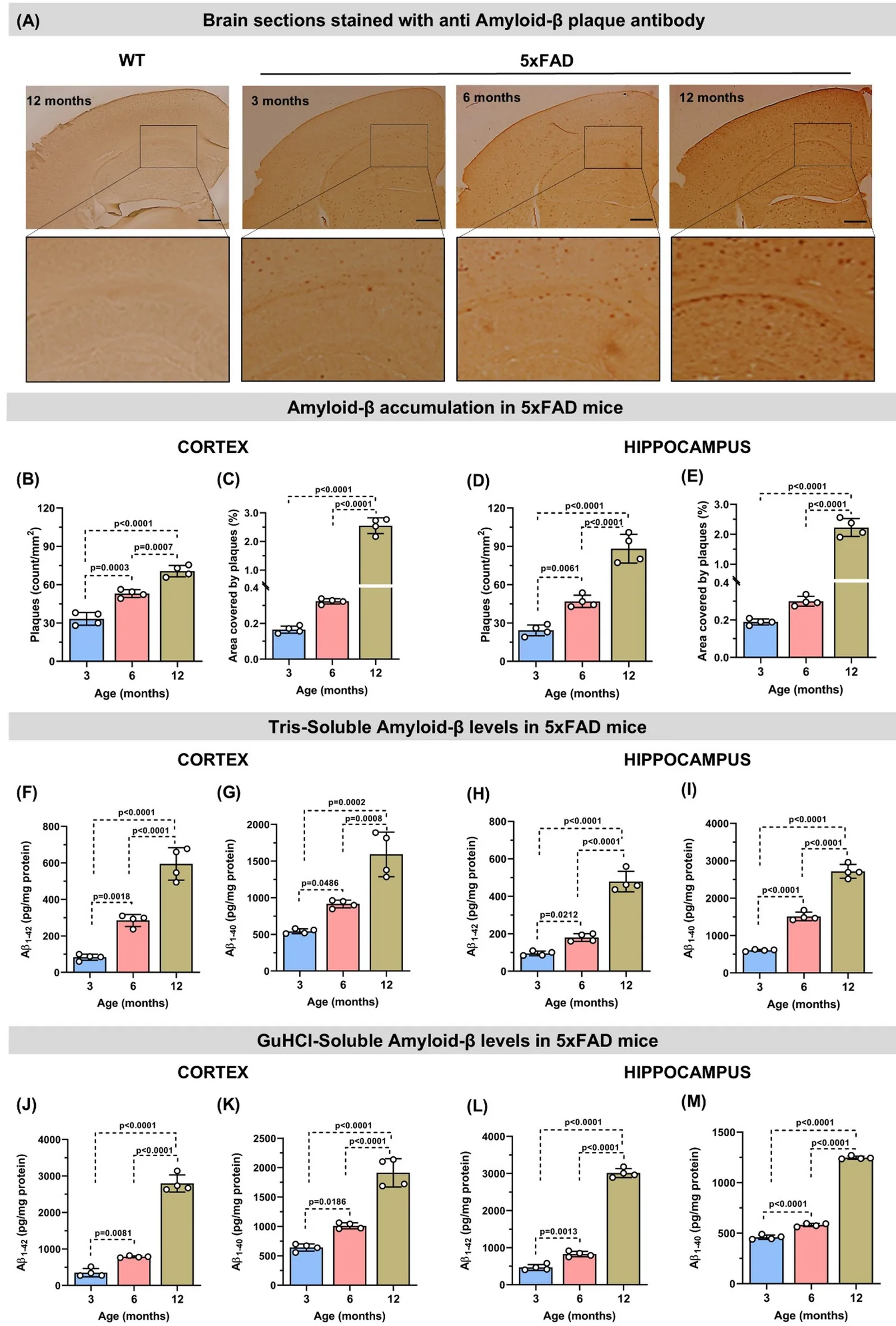
Age-dependent accumulation of amyloid species in 5xFAD mice. **A** Representative immunohistochemistry images of amyloid-β (Aβ) plaques in WT and 5xFAD mice at different ages. **B**-**E** Aβ-plaque count and burden in the cerebral cortex and hippocampus of 5xFAD mice across age groups. Levels of Tris-soluble Aβ_42_ and Aβ_40_ oligomers in the **F**,**G** cerebral cortex and **H**,**I** hippocampus of 5xFAD mice at different ages. Levels of GuHCl-soluble Aβ_42_ and Aβ_40_ in the **J**,**K** cerebral cortex and **L**,**M** hippocampus of 5xFAD mice. For immunohistochemical analysis, brain Sect. (6 per animal) from 5xFAD (*n* = 4) and WT (*n* = 4) mice were stained with an anti-amyloid antibody. Data are presented as mean ± SD, with individual points representing the average of 6 sections per animal. Statistical significance across age groups was determined using one-way ANOVA with Sidak’s multiple comparisons test. Scale bar: 1000 μm

To characterize the temporal evolution of amyloid pathology, Aβ deposition was quantified in 5xFAD mouse brains. Distinct Aβ plaques were detected both in the cerebral cortex and hippocampus of 5xFAD mice as early as 3 months and intensified with age (Fig. 2A). The cortical plaques increased from 24.3 ± 4.3 plaques/mm^2^ (*n* = 4) at 3 months to 70.6 ± 4.4 plaques/mm^2^ (*n* = 4) at 12 months (*p* = 0.005), with intermediate levels (53.0 ± 3.1 plaques/mm^2^, *n* = 4) at 6 months (Fig. 2B). The hippocampus followed a similar trajectory, with plaque count rising from 33.3 ± 5.0 plaques/mm^2^ (*n* = 4) at 3 months to 90.7 ± 8.9 plaques/mm^2^ (*n* = 4) at 12 months (*p* = 0.005). The age-dependent increase in Aβ-plaque load was accompanied by an expansion in fractional plaque area from ~ 0.17% at 3 months to ~ 2.5% at 12 months in both brain regions (Fig. 2C, E), suggesting progressive aggregation and spatial propagation of amyloid pathology.

To complement histological observations, soluble and aggregated Aβ species (Aβ_40_ and Aβ_42_) were quantified using ELISA in Tris-soluble and GuHCl-soluble brain fractions. The GuHCl fraction reflects highly aggregated yet soluble oligomeric Aβ species and does not exclusively represent insoluble plaques. Notably, both Aβ species were detected at 3 months and accumulated progressively in the cerebral cortex and hippocampus with age, mirroring Aβ plaques trends (Fig. 2F-M, Table S1). Interestingly, the Aβ_42_/Aβ_40_ ratio showed ~ 2.6-fold increase in the cerebral cortex from 3 to 12 months of age in 5xFAD mice (Table S1). However, the ratio increased only ~ 1.3 and ~ 1.6 fold for the Tris-soluble and GuHCl-soluble fractions, respectively, in the hippocampus. These data confirm the early onset and age-dependent escalation of pathogenic amyloid species that parallel cognitive decline in 5xFAD mice.

### Age-dependent neurometabolite alterations in 5xFAD mice

The neurometabolite homeostasis across disease stages was evaluated ex vivo in the cortical and hippocampal extracts from 5xFAD and WT mice. No significant differences (p_adj_≥0.303) in key neurometabolites were observed between genotypes at 3 and 6 months, suggesting a preserved neurometabolite homeostasis during the early stages of AD (Table S2). However, by 12 months, 5xFAD mice exhibited a marked reduction in NAA in both the cerebral cortex (5xFAD: 5.9 ± 0.4 µmol/g, *n* = 7, WT: 6.3 ± 0.2 µmol/g, *n* = 7; p_adj_=0.042) and hippocampus (5xFAD: 5.5 ± 0.6 µmol/g, *n* = 7; WT: 6.2 ± 0.5 µmol/g, *n* = 7; p_adj_=0.011) compared to WT controls (Table S2). In parallel, myo-inositol (m-Ino) was significantly elevated in these regions in the 5xFAD mice compared to WT mice (cortex: 7.6 ± 0.9 vs. 6.3 ± 1.2 µmol/g, p_adj_=0.028, hippocampus: 8.5 ± 1.1 vs. 7.4 ± 0.9 µmol/g, p_adj_=0.034). Similar to neurometabolite levels, NAA/m-Ino ratio, a screening marker of AD, was unaltered in the cerebral cortex (*p* = 0.231) and hippocampus (*p* = 0.130) of 5xFAD mice compared to WT mice at 3 months of age (Table S2). However, this ratio was significantly reduced at 6 months in both the cortex (-14%, 5xFAD: 0.81 ± 0.11, WT: 0.94 ± 0.15, *p* = 0.010) and hippocampus (-17%, 0.67 ± 0.08 vs. 0.80 ± 0.19, *p* = 0.028), and declined further by 12 months (cortex, -24%, *p* = 0.002; hippocampus, -23%, *p* = 0.001). Hence, the NAA/m-Ino ratio progressively declines with advancing AD pathology in 5xFAD mice and may serve as a better predictor of AD pathophysiology than individual metabolites.

### Stage-dependent neuronal metabolic impairment in 5xFAD mice

**Fig. 3:**
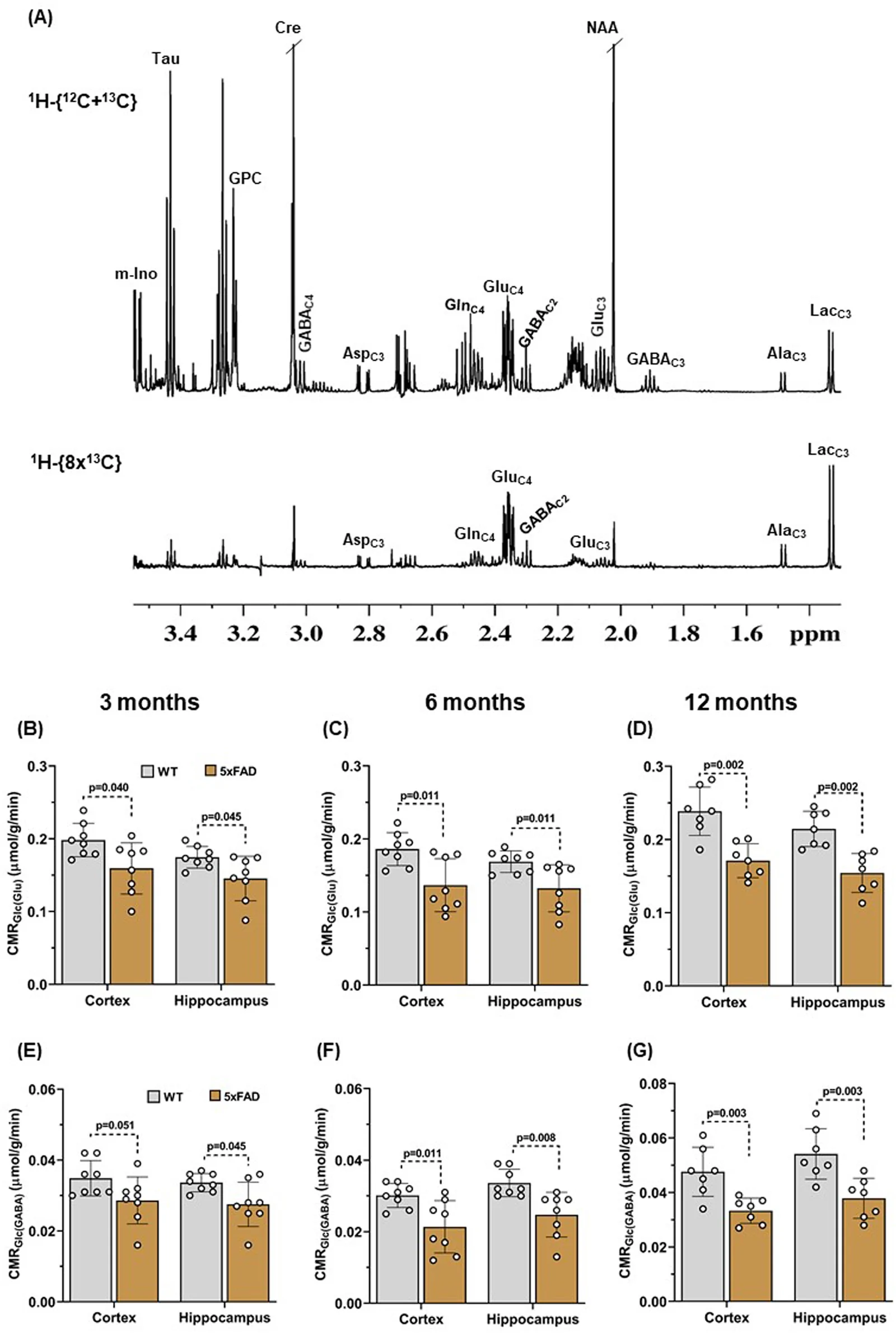
Age-dependent decline in neuronal glucose oxidation in 5xFAD mice. **A** Representative 1H-[^13^C]-NMR spectrum from hippocampal extract of 5xFAD mouse showing ^13^C labeled brain amino acids from [1,6-^13^C₂]glucose. Cerebral metabolic rates of glucose oxidation in **B**-**D** glutamatergic neurons (CMR_Glc(Glu)_) and **E**-**G** GABAergic neurons (CMR_Glc(GABA)_) in the cerebral cortex and hippocampus of 5xFAD (*n* = 7–8) and WT mice (*n* = 7–8) at 3, 6, and 12 months of age. Mice received an intravenous infusion of [1,6-^13^C₂]glucose for 10 min, and ^13^C labeling of amino acids was measured by ^1^H-[^13^C]-NMR spectroscopy in brain tissue extracts. CMR_Glc(Ox)_ was calculated using Eqs. 2,3 based on ^13^C label trapped into different amino acids. Statistical significance of the difference between 5xFAD and WT mice was evaluated using an unpaired Student’s T-test with Holm-Sidak’s method for correction of multiple comparisons. Data are presented as mean ± SD, with each point representing the measurement from an individual animal

To examine how AD progression affects neuronal metabolic activity, ^13^C incorporation into different brain metabolites from [1,6-^13^C₂]glucose was measured using ^1^H-[^13^C]-NMR spectroscopy (Fig. 3A). At 3 months, 5xFAD mice exhibited a significant reduction (p_adj_≤0.034) in ^13^C labeling of Asp_C3_ and Glu_C3_ in the cerebral cortex, and Asp_C3_ in the hippocampus (Table 1), indicating early impairment of TCA cycle activity. At 6 months, Asp_C3_ and Glu_C4_ labeling in the cortex and GABA_C2_, Gln_C4_, Glu_C3,_ and Glu_C4_ in the hippocampus were significantly decreased (p_adj_≤0.031). By 12 months, ^13^C labeling of all measured TCA cycle-linked amino acids was significantly reduced (p_adj_≤0.020) in both brain regions (Table 1), indicating widespread impairment of mitochondrial oxidative metabolism. The rates of glucose oxidation in the glutamatergic and GABAergic neurons were calculated using ^13^C trapped in these amino acids.

**Table 1:** Concentration (µmol/g) of ^13^C labeled amino acids from [1,6-^13^C_2_]glucose in 5xFAD and WT mice

Regions	Age	Groups	Glu_C4_	GABA_C2_	Gln_C4_	Asp_C3_	Glu_C3_	GABA_C4_
Cerebral Cortex	3 months	WT (*n* = 8)	1.87 ± 0.19	0.21 ± 0.02	0.20 ± 0.04	0.10 ± 0.02	0.29 ± 0.07	0.04 ± 0.02
		5xFAD (*n* = 8)	1.53 ± 0.34	0.17 ± 0.04	0.16 ± 0.05	0.07 ± 0.02*	0.19 ± 0.06*	0.04 ± 0.01
	6 months	WT (*n* = 8)	1.76 ± 0.22	0.17 ± 0.02	0.20 ± 0.05	0.10 ± 0.02	0.25 ± 0.07	0.04 ± 0.02
		5xFAD (*n* = 8)	1.30 ± 0.30**	0.13 ± 0.05	0.14 ± 0.05	0.06 ± 0.02**	0.19 ± 0.08	0.03 ± 0.01
	12 months	WT (*n* = 7)	2.05 ± 0.32	0.20 ± 0.04	0.26 ± 0.08	0.22 ± 0.04	0.31 ± 0.08	0.05 ± 0.02
		5xFAD (*n* = 7)	1.56 ± 0.27**	0.15 ± 0.02*	0.20 ± 0.05*	0.16 ± 0.04*	0.21 ± 0.06*	0.03 ± 0.01*
Hippocampus	3 months	WT (*n* = 8)	1.54±0.012	0.18 ± 0.01	0.18 ± 0.03	0.09 ± 0.01	0.25 ± 0.03	0.03 ± 0.01
		5xFAD (*n* = 8)	1.36 ± 0.28	0.16 ± 0.04	0.15 ± 0.04	0.07 ± 0.01*	0.19 ± 0.05	0.02 ± 0.01
	6 months	WT (*n* = 8)	1.55 ± 0.12	0.20 ± 0.01	0.19 ± 0.03	0.08 ± 0.01	0.23 ± 0.04	0.03 ± 0.01
		5xFAD (*n* = 8)	1.21 ± 0.29*	0.14 ± 0.03**	0.13 ± 0.04*	0.07 ± 0.02	0.17 ± 0.05*	0.02 ± 0.01
	12 months	WT (*n* = 7)	1.84 ± 0.22	0.27 ± 0.04	0.25 ± 0.06	0.19 ± 0.03	0.31 ± 0.05	0.04 ± 0.02
		5xFAD (*n* = 7)	1.33 ± 0.29**	0.19 ± 0.05*	0.19 ± 0.05*	0.14 ± 0.03*	0.21 ± 0.06*	0.02 ± 0.01**

Urethane (1.5 g/kg) anesthetized mice were infused with [1,6-^13^C_2_]glucose for 10 min, and ^13^C concentrations were measured from tissue extracts in ^13^C-edited spectra using [2-^13^C]glycine as an internal reference. Data are expressed as mean ± SD. Statistical significance between groups was assessed using Multiple unpaired t-tests with the Holm-Sidak method of multiple comparisons

*Abbreviations*: *Asp*_C3_ [3-^13^C]aspartate, *GABA*_C2_ [2-^13^C]GABA, *GABA*_C4_ [4-^13^C]GABA, *Glu*_C3_ [3-^13^C]glutamate, *Glu*_*C4*_ [4-^13^C]glutamate, *Gln*_C4_ [4-^13^C]glutamine

*p_adj_<0.05, **p_adj_<0.01 when 5xFAD was compared with WT mice

**Cerebral cortex:** At 3 months, there was a significant reduction (-22%, p_adj_=0.040) in the CMR_Glc(Glu)_ of 5xFAD mice (0.162±0.037 µmol/g/min, *n*=8) compared to WT mice (0.199±0.025 µmol/g/min, *n*=8; Fig. 3B). Further reductions in CMR_Glc(Glu)_ in 5xFAD mice were observed at 6 months (-25%, p_adj_=0.011) as well as 12 months (-28%, p_adj_=0.002; Fig. 3C, D). Similarly, CMR_Glc(GABA)_ in 5xFAD mice was markedly reduced at 3 months (-22%, p_adj_=0.051), 6 months (-27%, p_adj_=0.011), and 12 months (-32%, p_adj_=0.003; Fig. 3E–G).

**Hippocampus:** A similar pattern was observed in the hippocampus, where the CMR_Glc(Glu)_ in 5xFAD mice was reduced significantly (-16%, p_adj_=0.045) at 3 months compared to WT mice (Fig. 3B). This was further exacerbated at 6 months (-22%, p_adj_=0.011) and 12 months (-30%, p_adj_=0.003; Fig. 3C, D). Likewise, CMR_Glc(GABA)_ showed a reduction at 3 months (-18%, p_adj_=0.045), 6 months (-27%, p_adj_=0.008), and 12 months (-33%, p_adj_=0.003; Fig. 3E–G). 

These findings reveal early and progressive impairments in mitochondrial function in both glutamatergic and GABAergic neurons of 5xFAD mice, suggesting that bioenergetic insufficiency precedes overt symptoms.

### Progressive neurodegeneration in 5xFAD mice

**Fig. 4:**
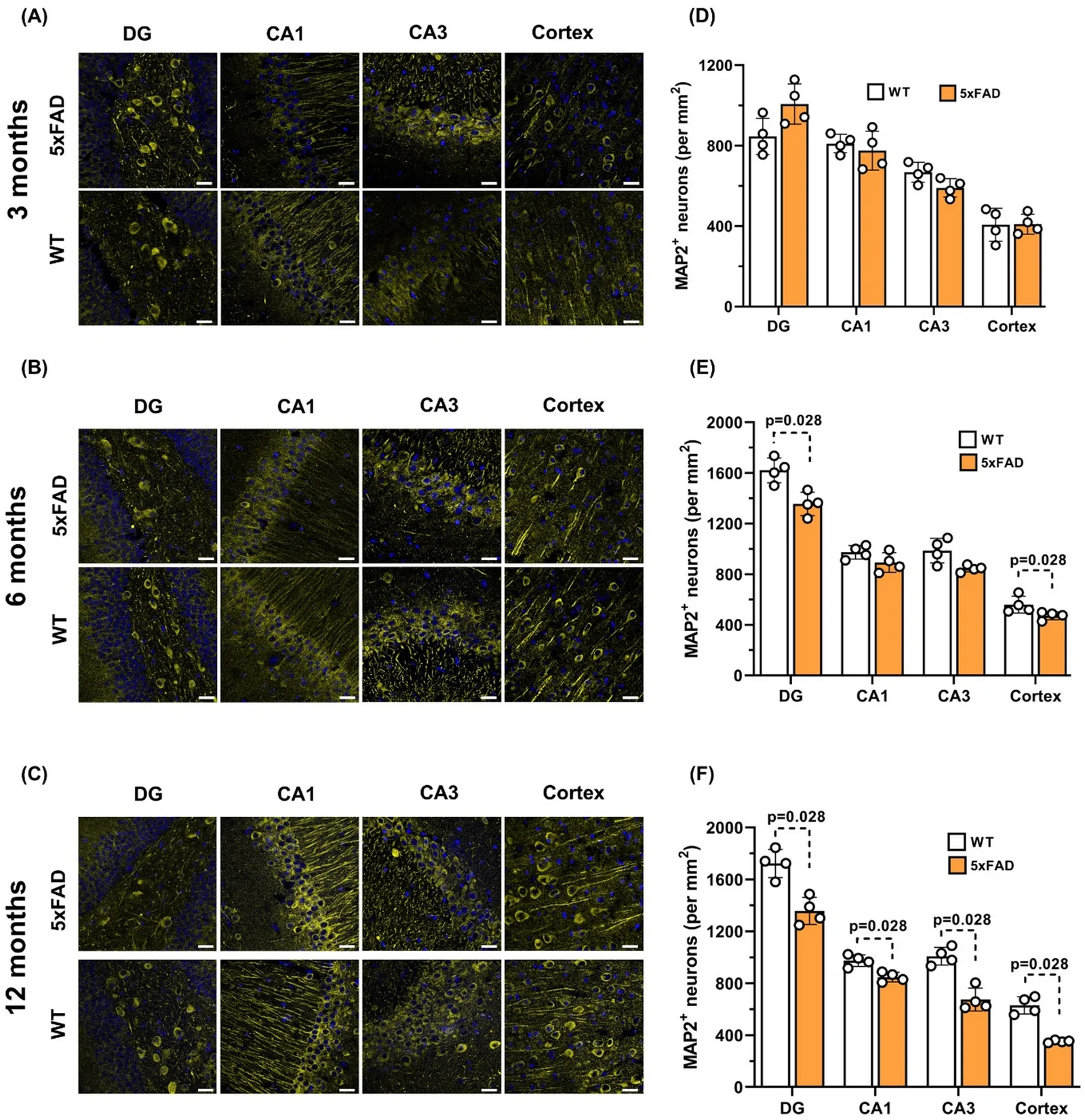
Age-dependent neuronal loss in 5xFAD mice. Representative confocal microscopy images (63x) showing MAP2+ neurons (yellow) in different brain regions of **A**-**C** 5xFAD (*n* = 4) and WT mice (*n* = 4). **D**-**F** Neuron count (per mm^2^) at different ages. Neurons were stained with anti-MAP2 antibody followed by Alexa 555 conjugated secondary antibody, and nuclei were stained using DAPI (blue). Each point represents the average measurements of 5–6 sections per animal. Data are presented as mean ± SD, and statistical significance of group difference was assessed using the Mann-Whitney U-test. Scale bar: 25 μm

To determine whether these metabolic deficits is due to functional impairment or actual neuronal loss, MAP2+ neurons were evaluated in the cerebral cortex and hippocampal subregions of 5xFAD (*n* = 4) and WT (*n* = 4) mice. At 3 months, neuronal density remained unchanged in 5xFAD mice (*p* ≥ 0.114; Fig. 4A, D), confirming preserved neuronal count despite early metabolic deficits. By 6 months, neuron count declined significantly (*p* = 0.028) in DG and cortex, with CA1 and CA3 regions showing a downward trend, suggesting region-specific neuronal loss (Fig. 4B, E). At 12 months, neuronal loss became more widespread, with a significant reduction (*p* = 0.028) in MAP2+ neurons across the hippocampus and cortex (Fig. 4C, F). Together with the neurometabolic data, these findings suggest neuronal metabolic impairment at the presymptomatic stage, followed by gradual neuronal loss and altered neuronal bioenergetics with advancing AD pathology.

### Upregulation of astroglial metabolism with disease progression

**Fig. 5:**
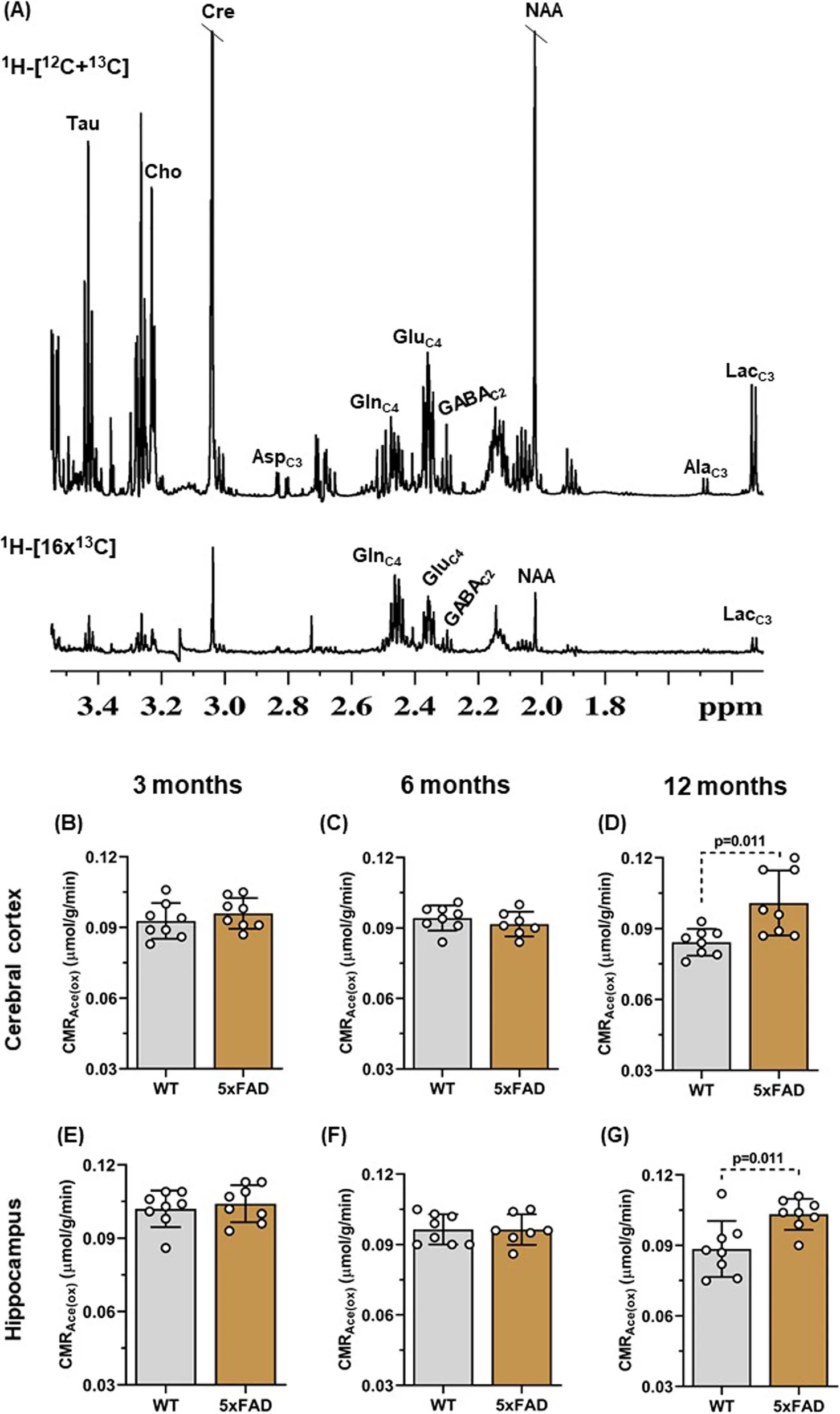
Age-dependent increase in astroglial metabolic activity in 5xFAD mice. **A** Representative ^1^H-[^13^C]-NMR spectrum from hippocampal extract showing ^13^C-labeling of brain amino acids from [2-^13^C]acetate. Cerebral metabolic rate of acetate oxidation (CMR_Ace(Ox)_) in the cerebral cortex and hippocampus **B**, **E** 3 months, **C**, **F** 6 months and **D**, **G** 12 months of age. Mice were intravenously infused with [2-^13^C]acetate for 15 min, and ^13^C labeling of amino acids was measured in brain tissue extracts using ^1^H-[^13^C]-NMR spectroscopy. CMR_Ace(Ox)_ was calculated from ^13^C trapped into amino acids using Eq. 4. Statistical significance of differences between 5xFAD (n = 7–8) and WT (n = 8) mice was evaluated using an unpaired Student’s T-test. Data are presented as mean ± SD, with each point representing the measurement from an individual mouse

**Fig. 6:**
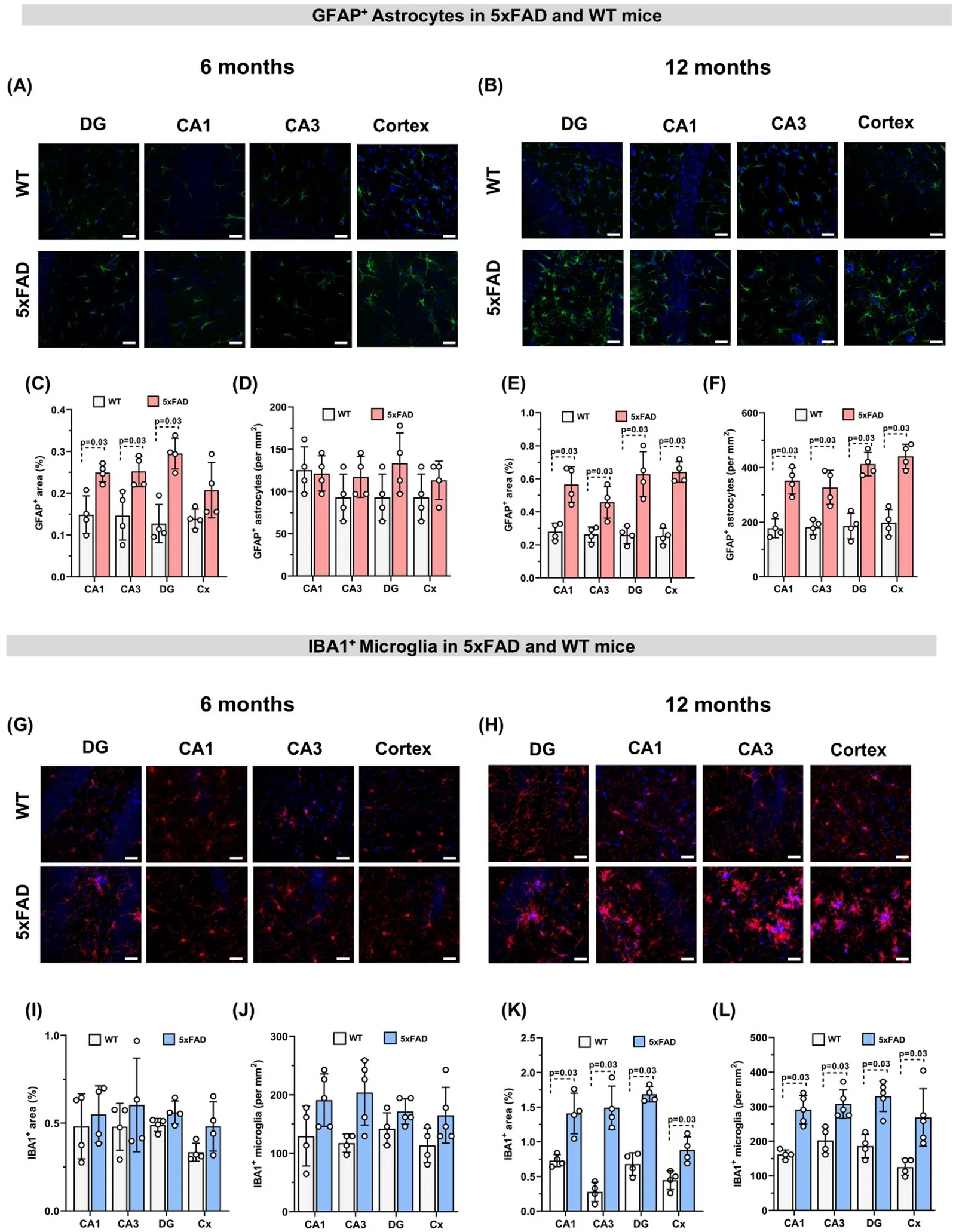
Age-dependent gliosis in 5xFAD mice. Representative confocal microscopy images (63×) showing **A**, **B** GFAP (green) and **G**, **H** IBA1 (red) immunostaining in the cerebral cortex and hippocampus at 6 and 12 months of age. Nuclei were stained with DAPI (blue). **C**-**F** GFAP+ area (%) and astrocyte count (per mm2) in 6- and 12-month-old 5xFAD (*n* = 4) and WT (*n* = 4) mice brains. **I**-**L** IBA1+ area (%) and microglia count (per mm^2^) in both brain regions. Brain sections were stained with anti-GFAP antibody conjugated with FITC for astrocytes and anti-IBA1 antibody, followed by Alexa 555 conjugated secondary antibody for microglia. Each dot represents the average measurement from 2–3 sections per animal. Data are presented as mean ± SD, and statistical significance of group difference was assessed using the Mann–Whitney U-test. Scale bar: 25 μm

Astroglia plays a central role in neurotransmitter clearance and recycling, thereby supporting neuronal signaling [34, 35]. The functional status of astrocytes in 5xFAD mice was assessed at 3, 6, and 12 months by following ^13^C-labeling of cerebral amino acids from [2-^13^C]-acetate (Fig. 5A). ^13^C-Labeling of cortical and hippocampal amino acids in 5xFAD mice was comparable to that of WT mice till 6 months (Table S3). However, at 12 months, 5xFAD mice exhibited increased cortical Gln_C4_ levels (0.57 ± 0.11 vs. 0.44 ± 0.05 µmol/g, p_adj_=0.032; Table S3) compared with WT mice. Consequently, the CMR_Ace(ox)_ was not significantly changed in the cortex (5xFAD: 0.096 ± 0.007 µmol/g/min, *n* = 8; WT: 0.093 ± 0.007 µmol/g/min, *n* = 8; *p* = 0.374) and hippocampus (0.104 ± 0.008 vs. 0.102 ± 0.007 µmol/g/min, *p* = 0.581) of 5xFAD mice at 3 months (Fig. 5B, E) and 6 months (cortex: 0.092 ± 0.005 vs. 0.094 ± 0.005 mol/g/min, *n* = 7–8, *p* = 0.371; hippocampus: 0.096 ± 0.006 vs. 0.097 ± 0.006 µmol/g/min, *n* = 7–8, *p* = 0.983; Fig. 5C, F). In contrast, at 12 months, CMR_Ace(ox)_ was significantly increased in both the cortex (5xFAD: 0.101 ± 0.014 µmol/g/min, *n* = 8; WT: 0.084 ± 0.006 µmol/g/min, *n* = 8; *p* = 0.011) and hippocampus (0.103 ± 0.006 vs. 0.089 ± 0.012 µmol/g/min, *p* = 0.011; Fig. 5D, G). These results indicate a stage-dependent increase in astrocytic activity, in contrast to the sustained decline in neuronal glucose oxidation.

### Progressive astroglial and microglial activation in 5xFAD mice

To elucidate the cellular basis underlying enhanced astroglial metabolism, glial morphology was investigated in 5xFAD mice at different ages. Astrocytic count (*p* ≥ 0.343) and area (*p* ≥ 0.286) were unaltered in the cortex and hippocampus of 3-month-old 5xFAD mice compared to WT mice (Fig. S4). However, a significant increase (*p* = 0.029) in the astrocytic area within the hippocampus at 6 months without a corresponding change in the cell number (Fig. 6A, C, D), suggesting hypertrophic remodeling rather than increased cell burden at this stage. By 12 months, both GFAP+ area and cell number were significantly increased (*p* = 0.029) in both the brain regions of 5xFAD mice (Fig. 6B, E, F), consistent with a transition from astrocytic hypertrophy to increased GFAP+ astrocyte burden and reactive gliosis as the disease advanced. Microglial morphology followed a similar temporal pattern. IBA1 staining revealed no significant change in area or cell number at 6 months (Fig. 6G, I, J), but both measures were markedly increased (*p* = 0.029) in the cerebral cortex and hippocampus at 12 months (Fig. 6H, K, L), reflecting microglial activation at the advanced stage of AD.

**Fig. 7:**
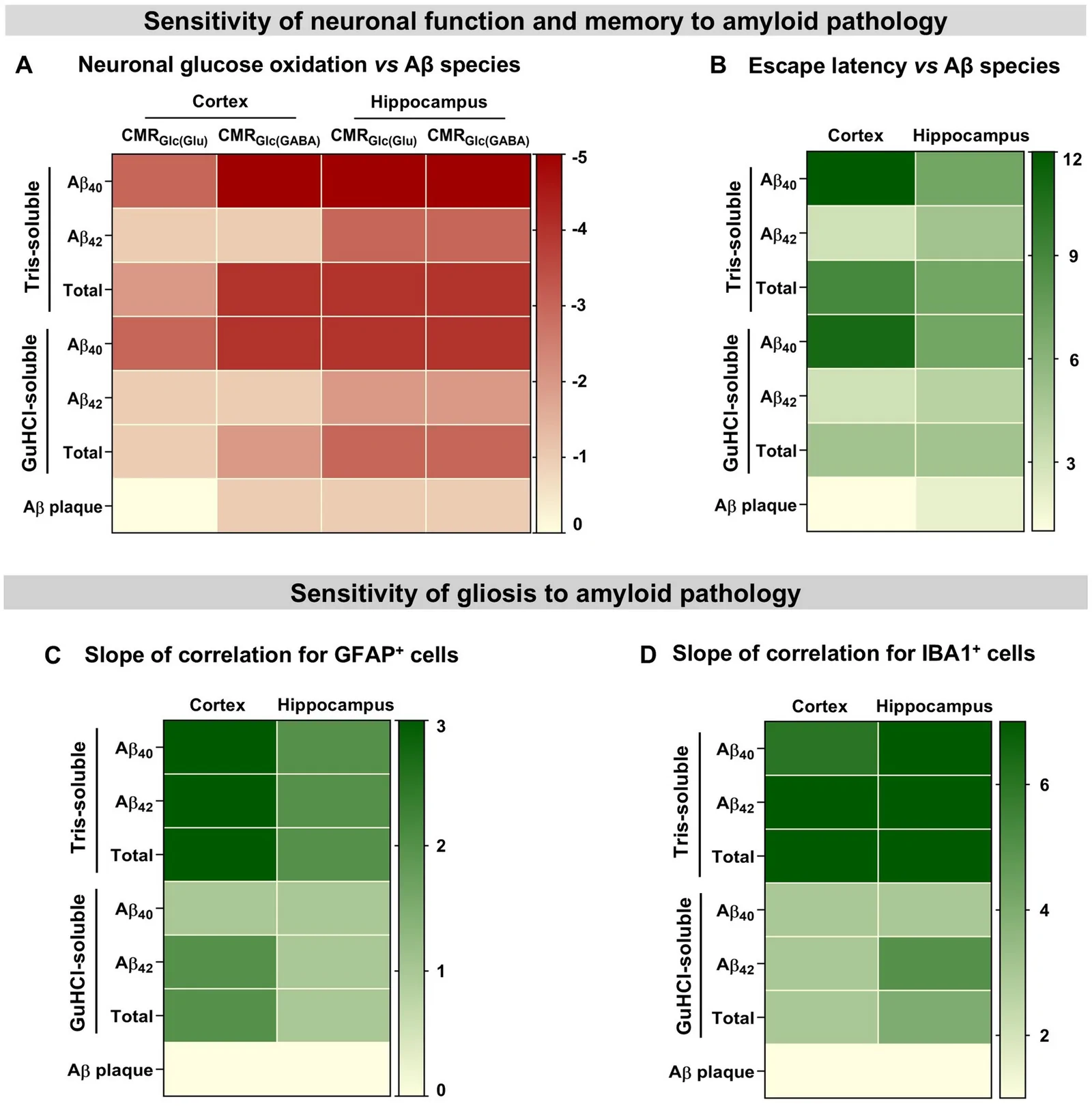
Sensitivity of neuronal metabolic impairment, memory deficit, and gliosis to individual amyloid species in 5xFAD mice. **A**, **B** Heat map depicting the sensitivity of impairment in neuronal glucose oxidation and memory to different Aβ species in the cerebral cortex and hippocampus of 5xFAD mice. Each cell represents the slope of the linear relationship between the respective measures and the corresponding amyloid species level. To facilitate comparisons across parameters, average Aβ levels were normalized to their respective 3-month values and used to estimate their impact on neurometabolism and cognitive function. **C**, **D** Heat map showing the sensitivity of gliosis to different Aβ species during AD progression. Each cell represents the slope of the linear relationship between the respective glial cells and the amyloid species, normalized to the 6-month age point

To further assess whether glial activation was associated with individual Aβ species or relative amyloid composition, correlation analyses were performed between glial cell burden and amyloid pathology. For this, the amyloid species levels and glial cell counts at 6 and 12 months of age were normalized to the corresponding 6-month values. The slopes of the linear relationship were used as an index of the sensitivity to glial activation. Quantitative analyses revealed that both GFAP+ and IBA1+ burdens were strongly associated with amyloid plaque load and soluble Aβ oligomers (Fig. S1). However, soluble Aβ_40_ oligomers showed higher sensitivity to enhanced glial pathology among individual species (Fig. 7C, D). Moreover, Aβ_42_/Aβ_40_ ratios showed higher sensitivity to GFAP+ and IBA1+ cells than the individual components (Fig. S2). Together, these findings suggest progressive glial remodeling in 5xFAD mice characterized by early astrocytic hypertrophy, followed by increased microglial recruitment. This progression parallels amyloid accumulation and coincides with the observed elevation in astroglial metabolic activity.

### Divergent metabolic-cognitive relationships at advanced-stage at 5xFAD mice

To explore the relationship between cell-specific metabolic activity and cognitive performance, correlations between MWM escape latency and cerebral metabolic fluxes were assessed in 5xFAD mice across disease stages. No significant correlations were observed between cognitive performance and neural metabolism up to the symptomatic stage (Fig. S3A-D). However, at the advanced stage, neuronal glucose oxidation showed a strong inverse correlation with the escape latency in the cortex (Glutamatergic neurons: *r*=-0.753, *p* = 0.003; GABAergic neurons: *r*=-0.581, *p* = 0.037) and hippocampus (Glutamatergic neurons: *r*=-0.666, *p* = 0.013; GABAergic neurons: *r*=-0.623, *p* = 0.034; Fig. S3E, F). These findings suggest that reduced neuronal oxidative metabolism is closely associated with memory impairment at the advanced AD stage. In contrast, CMR_Ace(ox)_ positively correlated with escape latency in both cerebral cortex (*r* = 0.540, *p* = 0.038) and hippocampus (*r* = 0.544, *p* = 0.036) at 12 months (Fig. S3G, H), suggesting that increased astroglial activity may reflect a reactive response to escalating amyloid pathology, potentially contributing to neuronal metabolic dysfunction.

### Neuronal metabolic impairment is associated with soluble Aβ_40_ oligomers

To identify which amyloid species affect neuronal metabolic decline, the relationship between age-dependent increase in Aβ species and reduction in glucose oxidation in excitatory and inhibitory neurons was examined. For this analysis, average cortical and hippocampal Aβ levels at 6 and 12 months were normalized to their 3-month levels and used to estimate their impact on neurometabolic impairment. The slope of the line represented the sensitivity of each amyloid component to metabolic impairment. In the cortex, both Tris-soluble and GuHCl-soluble Aβ_40_ oligomers exhibited high sensitivity for reductions in the metabolic activity of glutamatergic neurons (slope ~ -3) as well as GABAergic neurons (slope ~ -3 to -5; Fig. 7A). A similar or even greater impact of Aβ_40_ oligomers (slope ~ -4 to -5) was seen in the hippocampus. In contrast, Aβ_42_ exhibited minimal effect on the neurometabolic activity in both neuronal types (slope ~ -1 to -2). Additionally, plaque burden, despite a drastic increase (≥ 11 fold) from 3 to 12 months, showed a negligible or minimal impact (slope ~ 0 to -1) on neuronal metabolic impairment (Fig. 7A).

The sensitivity pattern of amyloid species to memory closely paralleled the neurometabolic trends: Tris- and GuHCl-soluble Aβ_40_ oligomers in both the cerebral cortex (slope ~ 11 to 12) and hippocampus (slope ~ 7) showed the greatest impact on escape latency in the MWM test (Fig. 7B). In contrast, plaque burden and Aβ_42_ oligomers, displayed minimal impact (slope ~ 1 to 4) to escape latency. These findings provide in vivo evidence that among individual Aβ species, Aβ_40_ oligomers exhibit higher sensitivity to neurometabolic dysfunction and cognitive decline.

The sensitivity of impairment in neuronal metabolic activity and cognitive function to the Aβ_42_/Aβ_40_ ratio was also examined. The Aβ_42_/Aβ_40_ ratio showed lower sensitivity to neurometabolic impairment in the cerebral cortex and relatively higher sensitivity for the hippocampus. A similar pattern was also noted for memory decline (Fig. S2A, B).

## Discussion

The present study provides a quantitative, cell-type-resolved view of brain energy metabolism during AD progression in 5xFAD mice. Although cerebral glucose hypometabolism is well recognized in AD, its cell-specific oxidative fate remains poorly understood. By directly measuring neuronal and astrocytic oxidative metabolic capacity across disease stages, this study revealed that neuronal glucose oxidation is impaired as early as the presymptomatic stage and remains persistently reduced. In contrast, astrocytic oxidative metabolism increased only at the advanced AD stage, coinciding with microgliosis. These findings indicate a sustained neuronal energetic failure that precedes neurodegeneration.

Cognitive performance in 5xFAD mice declined progressively, paralleling the temporal evolution of amyloid pathology. Learning and memory were preserved at 3 months, began to deteriorate by 6 months, and showed pronounced impairment at 12 months, mirroring the transition from soluble Aβ oligomers accumulation to extensive plaque deposition [17, 36]. Quantitative histopathological analyses revealed approximately 10-15-fold increase in Aβ plaque area in cortical and hippocampal regions between 3 and 12 months, reflecting the accelerated amyloidogenic profile characteristic of this model [18, 37]. However, consistent with observations in human subjects [38, 39] and rodent models [40, 41], early cognitive preservation despite a significant plaque burden suggests that insoluble aggregates alone may not be sufficient to cause functional decline. Accumulating evidence suggests that soluble Aβ oligomers, particularly low-molecular-weight protofibrils and mixed Aβ_42_/Aβ_40_ assemblies, are associated with impairment of synaptic signaling and mitochondrial integrity before plaque formation [42–44]. In line with this, the biochemical analyses in the current study revealed marked increases in both Aβ_42_ (~ 5-fold) and Aβ_40_ (> 3-fold) oligomers across disease stages, with soluble Aβ_40_ oligomers showing the highest impact on cognitive decline (Fig. 7B). While these data do not establish causality, they support a model in which diffusible Aβ_40_ oligomers track closely with early impairment of neuronal function.

^1^H MRS studies in mouse models [15, 45] and AD patients [46, 47] have reported perturbations in neurometabolite homeostasis with AD progression. In agreement with these reports, a reduction in NAA levels was observed in the cerebral cortex and hippocampus of 12-month-old 5xFAD mice. The reduced MAP2+ neuron counts in these brain regions (Fig. 4) accompanied a reduction in NAA levels, supporting the interpretation that late-stage disruption in neurometabolite homeostasis coincides with neurodegeneration. Myo-inositol, a known marker of astroglia [48], was markedly elevated in both regions, consistent with enhanced gliosis. Interestingly, although neurometabolite levels were largely preserved till 6 months in 5xFAD, the NAA/m-Ino ratio, a screening marker of AD, was reduced in the cerebral cortex (-14%) and hippocampus (-17%), suggesting a subthreshold neurometabolite imbalance associated with the onset of neuronal loss. This ratio declined further by 12 months (~-24%), reflecting progressive neurodegeneration accompanied by astrocytic activation. Together, these findings suggest that neurometabolite imbalance serves as a dynamic indicator of AD stage, reflecting the evolving balance between neuronal viability and glial activation throughout AD progression.

Brain glucose hypometabolism is a well-recognized hallmark of AD and is closely linked to compromised synaptic activity, and enhanced Aβ formation and aggregation [49]. FDG-PET studies have reported glucose hypometabolism in the posterior cingulate, occipital cortices, and lateral temporoparietal brain regions of subjects with mild cognitive impairment and AD patients [50, 51]. ^13^C NMR-based neurometabolic flux analysis in the current study revealed profound deficits in neuronal glucose oxidation rates of cortical and hippocampal neurons in 5xFAD mice as early as 3 months, with deficits deteriorating across AD progression (Fig. 3). These findings extend the prior ex vivo NMR reports of TCA cycle impairment in AD rodent models [12, 15, 52] by demonstrating that neuronal oxidative metabolism is compromised before neurodegeneration or overt cognitive decline. Emerging evidence suggests that cerebral glucose hypometabolism in AD may arise from disturbances at multiple levels of cellular energy metabolism, including altered glycolytic processing and impaired oxidative metabolism [53, 54]. Although these processes were not directly assessed in the present study, the observed reduction in neuronal glucose oxidation is consistent with the broader metabolic dysfunction reported in AD [55–57].

The energy generated through brain metabolism supports processes such as synaptic transmission, action potential generation, and the maintenance of resting membrane potentials [58]. As revealed from [2-^13^C]acetate oxidation, the astroglial activity is not significantly altered till the 6-month age (Fig. 5). Therefore, the reduction in amino acid labeling from [1,6-^13^C_2_]glucose at 3 and 6 months clearly indicates energy deficits in glutamatergic and GABAergic neurons in 5xFAD mice (Fig. 3; Table 1). This interpretation is consistent with the reports of reduced glucose metabolism in hippocampal slices measured using ^13^C-turnover approach, as well as reduced oxygen consumption rates measured in synaptosomes isolated from the hippocampus using Seahorse Bioanalyzer in 5xFAD mice at both 2 and 4 months of age [18].

Neuronal glucose oxidation and neurotransmitter cycle flux are reported to be stoichiometrically coupled in a 1:1 relationship across the full range of brain activity [59, 60]. Preservation of this coupling has also been reported in 12-month-old AβPP/PS1 mice, exhibiting extensive Aβ plaque burden and enhanced astroglial activity [15]. Thus, the observed reduction in glucose oxidation rates in glutamatergic and GABAergic neurons likely reflects reduced glutamatergic and GABAergic neurotransmission in 5xFAD mice. This is further supported by the reduced Gln_C4_ labeling from [1,6-^13^C_2_]glucose observed in the hippocampus of 5xFAD mice at 6 months of age (Table 1). The basal glutamate levels, as well as potassium chloride-evoked glutamate release, were enhanced in hippocampal subregions of AβPP/PS1 at different ages [61]. Additionally, excitatory-to-inhibitory imbalance is reported in the CA1 region in 5xFAD mice at the prodromal stage of AD [62]. Moreover, electrophysiological measurements indicated increased spontaneous excitatory postsynaptic currents in the CA1 region of the hippocampus, suggesting that enhanced excitatory activity may be localized to this subregion [18]. The divergence between neurometabolic and electrophysiological measurements in the hippocampus is likely attributable to the fact that electrophysiological recordings provide localized information from specific subregions, whereas neurometabolic measurements reflect neuronal activity of the entire hippocampus. At 12 months of age, both the cerebral cortex and hippocampus exhibited increased rates of acetate oxidation, suggesting enhanced metabolic activity of astrocytes and microglia. Therefore, the marked reduction in glucose oxidation rates observed in 5xFAD mice likely represents an even greater impairment in neuronal energetics than these values. Moreover, the neurotransmitter cycling flux, which is proportional to Gln_C4_ labeling, was decreased in the cerebral cortex and hippocampus of these mice.

Neurometabolic impairment becomes functionally limited with the progression of AD. Despite early metabolic deficits, cognitive impairment emerged at 6 months, with a strong negative correlation between neuronal glucose oxidation rate and escape latency by 12 months (Fig. S3), suggesting declining brain energy increasingly constrains cognitive function as AD pathology advances. This temporal dissociation implies that early energetic deficits do not immediately translate into behavioral impairment, whereas at later stages, when neurodegeneration and synaptic loss are more pronounced, neurometabolic failure becomes a limiting factor for cognitive performance. This pattern mirrors longitudinal PET reports in humans, in which regional glucose hypometabolism often precedes overt cognitive symptoms by years [63, 64], supporting the view that neuronal energetic impairment represents an early marker of vulnerability that acquires functional relevance as disease burden accumulates.

Astrocytes are central regulators of synaptic function and neuronal activity, and their transition to a reactive state is known to be involved in AD-related neuroinflammation [65, 66]. Beyond their inflammatory roles, astrocytes are also critical for neurotransmitter clearance and cycling, thereby maintaining neuron-astrocyte metabolic coupling. Disruption of this interaction has been implicated in AD pathogenesis and may contribute to impaired neuronal bioenergetics and neurotransmitter homeostasis [12, 18]. Furthermore, neuronal loss in AD has been linked to dysregulation of the glutamatergic system, a process that involves excitotoxicity resulting from impaired glutamate clearance from the synaptic cleft [67]. Downregulation of genes encoding glutamate and GABA receptors and transporters suggests that astrocytes may lose their synapse-supporting roles in AD, thereby actively contributing to synaptic dysfunction [68]. Consistent with this notion, inhibition of astrocyte reactivity improved learning and alleviated synaptic deficits in the hippocampus of AD models, further supporting a role for astrocytes in synaptic dysfunction in AD [69].

According to the astrocyte-neuron metabolic coupling model, neuronal activity stimulates astrocytic glucose uptake and glycolysis, and the pyruvate/lactate produced is sent to neurons to help meet their energy demands through metabolite exchange and neurotransmitter recycling [70, 71]. Acetate oxidation in the current study was not significantly altered in 5xFAD mice up to 6 months of age but was markedly enhanced at 12 months in both the cortex and hippocampus, suggesting increased astroglial metabolic activity at advanced AD stage (Fig. 5). In contrast, neuronal-astrocytic interaction, as revealed by Gln_C4_ labeling from [1,6-^13^C_2_]glucose (Table 1) and neuronal metabolic activity (Fig. 3), is markedly reduced from the symptomatic AD stage. This temporal pattern suggests that astrocytes undergo metabolic reprogramming at advanced stages, coinciding with amyloid accumulation and neurodegeneration.

The strong positive correlation between CMR_Ace(Ox)_ and escape latency (Fig. S3G, H) suggests that astroglial oxidative metabolism parallels cognitive deterioration. While this association does not imply causality, it suggests that astrocytic metabolic reprogramming may be due to sustained neuronal energy failure with AD progression. Immunohistochemical analyses revealed astrocytic hypertrophy and increased IBA1+ microglial burden, localized in the hippocampus at the symptomatic stage and generalized at the advanced AD stage, further supporting this interpretation. Notably, the strong associations noted between GFAP burden, soluble Aβ species, and Aβ_42_/Aβ_40_ ratios further support a close relationship between amyloid pathology and astroglial remodeling, particularly in the cortex. These findings are consistent with previous reports demonstrating tight spatial and biochemical coupling between amyloid accumulation and reactive gliosis in AD [72, 73]. Moreover, together with the sustained decrease in the NAA/m-Ino ratio, these data suggest that astrocytic metabolic activation and neuroinflammatory remodeling constitute an integrated response to advancing AD pathology, thereby reinforcing neurometabolic imbalance at the advanced disease stage.

Given the parallel progression of neuronal metabolic impairment and amyloid accumulation, the impact of different amyloid species on the deficit in neuronal energetics was examined. Despite limited sample resolution across disease stages, the age-dependent patterns were consistent across brain regions. Among the individual Aβ species examined, soluble Aβ_40_ oligomers showed the greatest sensitivity to reduced neuronal glucose oxidation and memory impairment, particularly in the hippocampus, a region critical for memory encoding (Fig. 7A, B). In contrast, Aβ_42_ oligomers and plaque burden exhibited the least sensitivity. Interestingly, the Aβ_42_/Aβ_40_ ratio, an indicator of amyloid composition, showed even greater sensitivity to neurometabolic impairment and memory deficits, particularly in the hippocampus. This may be attributable to the substantially narrower range of variation of the Aβ_42_/Aβ_40_ ratio than individual amyloid species across disease progression. Consequently, the greater sensitivity likely reflects the normalization inherent to the sensitivity analysis rather than a greater pathological influence of the ratio itself. Overall, these findings suggest that, among the individual species, Aβ_40_ oligomers exhibit the highest sensitivity to neuronal metabolic, behavioral, and glial alterations. While these relationships are associative and do not permit causal inference, they are consistent with prior clinical and experimental reports implicating soluble Aβ_40_ oligomers in metabolic and synaptic dysfunction [74, 75]. Within this context, the present findings position soluble Aβ_40_ oligomers as a candidate marker of neurometabolic failure that closely tracks with neuronal oxidative metabolic impairment and cognitive decline, rather than with plaque burden.

There are a few limitations associated with the current study. First, to avoid complications arising from female hormones, only male mice were used to assess the impact of AD progression on brain energy metabolism and cognitive function, which limits the generalizability of the findings to females. Second, due to technical constraints, the impact of amyloid pathology on cerebral glucose oxidation rates was assessed using group averages across age points rather than individual-level data, thereby limiting the scope of statistical evaluation. Consequently, the sensitivity analyses presented in this study should be considered descriptive measures rather than formal effect-size estimates, and direct quantitative comparisons of sensitivity across different amyloid biomarkers should be interpreted with caution. Nevertheless, these findings advance an integrative metabolic framework in which disrupted brain energy metabolism serves as a central axis linking amyloid pathology, glial remodeling, and cognitive decline in AD. Furthermore, because acetate is a non-physiological substrate and is utilized at substantially lower rates than glucose, metabolic measurements using ^13^C-labeled acetate are less sensitive to subtle astroglial alterations than glucose-derived neurometabolic analysis. Hence, the absence of significant changes in metabolite labeling from [2-^13^C]acetate and in CMR_Ace(ox)_ does not necessarily indicate completely unaltered astroglial activity. However, the largely unchanged GFAP+ and IBA+ cells in the cerebral cortex suggest that astroglial function remains relatively preserved in 5xFAD mice up to 6 months of age. Finally, the study identifies the sensitivity of soluble Aβ oligomers to multiple cellular and physiological measures, including metabolism, gliosis, and behavior, but does not establish a causal relationship. However, previous reports demonstrating the toxicity of soluble Aβ oligomers in cell lines and mixed neuron-glia cultures [76, 77] suggest that these findings may have implications beyond simple correlation and need further investigations.

## Conclusions

Collectively, this study delineates a temporal neurometabolic framework in which progressive amyloid accumulation is associated with progressive impairment in neuronal metabolic activity, followed by cognitive decline, while astroglial metabolic engagement and reactive gliosis emerge at later stages of disease progression. This temporal alignment among Aβ_40_ oligomers, neuronal hypometabolism, and cognitive decline supports a model in which diffusible amyloid species are closely linked to early energetic stress in vulnerable neuronal populations.

## Supplementary Information

Supplementary Material 1.

## Data Availability

The datasets generated in the current study are available from the corresponding author on reasonable request.

## Abbreviations

Ala: Alanine
Ala_C3_: [3-^13^C]alanine
Asp: aspartate
Asp_C3_: [3-^13^C]aspartate
Cho: Choline
CNS: Central nervous system
CMR_Ace(Ox)_: Cerebral metabolic rate of acetate oxidation
CMR_Glc(GABA)_: Cerebral metabolic rate of glucose oxidation in GABAergic neurons
CMR_Glc(Glu)_: Cerebral metabolic rate of glucose oxidation in glutamatergic neurons
CMR_Glc(Ox)_: Cerebral metabolic rate of glucose oxidation
GABA: γ-aminobutyric acid
GABA_C2_: [2-^13^C]GABA
GABA_C4_: [4-^13^C]GABA
Gln: Glutamine
Gln_C4_: [4-^13^C]glutamine
Glu: Glutamate
Glu_C3_: [3-^13^C]glutamate
Glu_C4_: [4-^13^C]glutamate
m-Ino: Myo-inositol
NAA: N-acetylaspartate
NMR: Nuclear magnetic resonance
TSP: 3-trimethylsilyl[2,2,3,3-D_4_]-propionate
