# Supplementary material for: In vivo evidence for soluble Aβ oligomer-driven neuronal metabolic dysfunction in 5xFAD mice
Source: Alzheimers Res Ther. 2026 Jul 11;18:235. doi: 10.1186/s13195-026-02130-4 (PMC13700184; doi:10.1186/s13195-026-02130-4)

Supporting Information

In Vivo Evidence for Soluble Aβ Oligomer-Driven Neuronal Dysfunction in 5xFAD Mice

Chaynita Dashora[1,2] and Anant Bahadur Patel[1,2]*

[1]NMR Microimaging and Spectroscopy, CSIR-Centre for Cellular and Molecular Biology, Habsiguda, Uppal Road, Hyderabad 500007, India; [2]Academy of Scientific and Innovative Research (AcSIR), Ghaziabad 201002, India

* Address for Correspondence:
Dr. Anant B. Patel, NMR Microimaging and Spectroscopy, Centre for Cellular and Molecular Biology, Uppal Road, Habsiguda, Hyderabad 500 007, India

Telephone: +91-40-27192838

Email: abpatel@ccmb.res.in; abpatel@csirccmb.org

Running Headline: Neuronal–Glial Metabolic Dynamics in Familial AD

Abbreviations

Ala, alanine; Ala_{C3}, [3-^{13}C]alanine; Asp, aspartate; Asp_{C3}, [3-^{13}C]aspartate; Cho, choline; CNS, central nervous system; $CMR_{Ace(Ox)}$, cerebral metabolic rate of acetate oxidation; $CMR_{Glc(GABA)}$, cerebral metabolic rate of glucose oxidation in GABAergic neurons; $CMR_{Glc(Glu)}$, cerebral metabolic rate of glucose oxidation in glutamatergic neurons; $CMR_{Glc(Ox)}$, cerebral metabolic rate of glucose oxidation; GABA, γ-aminobutyric acid; $GABA_{C2}$, [2-^{13}C]GABA; $GABA_{C4}$, [4-^{13}C]GABA; Gln, glutamine; Gln_{C4}, [4-^{13}C]glutamine; Glu, glutamate; Glu_{C3}, [3-^{13}C]glutamate; Glu_{C4}, [4-^{13}C]glutamate; m-Ino, myo-inositol; NAA, N-acetylaspartate; NMR, nuclear magnetic resonance; TSP, 3-trimethylsilyl[2,2,3,3-D_4]-propionate.

Table S1 Human Aβ levels in brain regions of 5xFAD mice with age

Region	Age	$A\beta_{42}$	$A\beta_{40}$	$A\beta_{42}/A\beta_{40}$
**Cerebral Cortex**	**Tris-soluble oligomers (pg/mg total protein)**			
	3 months (n=4)	78.8±10.8	543.4±33.5	0.15±0.03
	6 months (n=4)	327.5±10.0**	916.2±53.6*	0.36±0.02****
	12 months (n=4)	636.7±46.5****	1592.6±304.1****	0.41±0.05****
	**GuHCl-soluble oligomers (pg/mg total protein)**			
	3 months (n=4)	351.4±114.8	641.9±61.2	0.52±0.17
	6 months (n=4)	935.3±7.9**	1009.9±51.3*	1.00±0.04***
	12 months (n=4)	2793.6±233.4****	1913.7±241.4****	1.35±0.10****
**Hippocampus**	**Tris-soluble oligomers (pg/mg total protein)**			
	3 months (n=4)	96.7±11.1	604.6±25.1	0.15±0.02
	6 months (n=4)	246.1±37.2*	1511±114.9****	0.18±0.02
	12 months (n=4)	515.3±31.1****	2716±185.0****	0.19±0.00*
	**GuHCl-soluble oligomers (pg/mg total protein)**			
	3 months (n=4)	467.2±80.1	449.3±29.2	1.29±0.22
	6 months (n=4)	828.3±72.3**	581.9±15.8****	1.86±0.17**
	12 months (n=4)	3011.7±122.8****	1247.3±15.7****	2.07±0.09***

Levels of Tris- and Guanidine HCl (GuHCl)-soluble $A\beta_{40}$ and $A\beta_{42}$ oligomers were quantified using specific ELISA kits. The data are presented as mean±SD. Statistical significance between groups was assessed using One-way ANOVA with Sidak's multiple comparisons. *p<0.05, **p<0.01, ***p<0.001, ****p<0.0001 when compared to 3-month-old mice.

Table S2 Concentration (μmol/g) of neurometabolites in 5xFAD and WT mice

Regions	Age	Groups	Glu	GABA	Gln	Asp	NAA	m-Ino	NAA/m-Ino
Cerebral Cortex	3 months	WT (n=8)	12.4±0.5	3.0±0.3	4.3±0.2	1.7±0.1	6.6±0.4	8.1±0.3	0.87±0.11
		5xFAD (n=8)	12.0±0.7	2.8±0.4	4.3±0.4	1.7±0.1	6.8±0.4	7.8±0.3	0.91±0.07
	6 months	WT (n=8)	12.2±1.2	2.5±0.3	5.2±1.6	2.2±0.7	6.5±0.6	7.0±1.1	0.94±0.15
		5xFAD (n=8)	12.1±1.2	2.6±0.2	4.8±0.6	2.2±0.5	6.2±1.1	7.7±0.6	0.81±0.11*
	12 months	WT (n=7)	12.0±0.7	2.4±0.2	5.1±1.2	3.0±0.3	6.3±0.2	6.3±1.2	1.03±0.19
		5xFAD (n=7)	11.7±0.7	2.6±0.3	4.9±0.4	2.8±0.3	5.9±0.4*	7.6±0.9*	0.78±0.10**
Hippocampus	3 months	WT (n=8)	11.8±0.5	3.0±0.3	4.3±0.2	1.5±0.1	6.1±0.2	9.7±0.7	0.69±0.08
		5xFAD (n=8)	11.3±0.4	3.0±0.4	4.2±0.3	1.4±0.1	6.1±0.4	8.7±0.3	0.72±0.04
	6 months	WT (n=8)	11.3±1.1	2.9±0.5	5.2±2.0	1.8±0.5	5.9±0.4	7.6±1.1	0.80±0.19
		5xFAD (n=8)	11.4±0.9	2.8±0.3	4.4±0.5	1.9±0.5	5.5±0.7	8.2±0.3	0.67±0.08*
	12 months	WT (n=7)	11.7±0.6	2.8±0.2	5.0±1.2	2.5±0.3	6.2±0.5	7.4±0.9	0.85±0.13
		5xFAD (n=7)	11.8±0.6	2.8±0.3	4.6±0.4	2.4±0.4	5.5±0.6*	8.5±1.1*	0.66±0.12**

The concentration of neurometabolites was measured in tissue extracts in the non-edited ^{1}H-[^{13}C]-NMR spectrum using [2-^{13}C]glycine as reference. Values are presented as mean±SD. Statistical significance was determined using unpaired t-tests with the Holm-Sidak method of correction for multiple comparisons. *$p_{adj}<0.05$ when 5xFAD mice were compared with WT mice. Abbreviations: Asp, Aspartate; GABA, γ-Amino Butyric Acid; Glu, Glutamate; Gln, Glutamine; m-Ino, myo-Inositol; NAA, N-Acetyl Aspartate.

Table S3 Concentration (μmol/g) of ^{13}C labeled metabolites from [2-^{13}C]acetate in 5xFAD and WT mice

Regions	Age	Groups	Glu_{C4}	$GABA_{C2}$	Gln_{C4}	Asp_{C3}	Glu_{C3}	$GABA_{C4}$
**Cerebral Cortex**	3 months	WT (n=8)	0.45±0.04	0.06±0.00	0.51±0.04	0.02±0.00	0.15±0.03	0.02±0.01
		5xFAD (n=8)	0.47±0.03	0.06±0.01	0.52±0.03	0.03±0.00	0.16±0.02	0.02±0.01
	6 months	WT (n=8)	0.42±0.04	0.06±0.01	0.48±0.04	0.04±0.00	0.17±0.01	0.02±0.00
		5xFAD (n=7)	0.42±0.02	0.06±0.01	0.48±0.04	0.04±0.00	0.15±0.01	0.02±0.00
	12 months	WT (n=8)	0.38±0.05	0.05±0.01	0.44±0.05	0.04±0.01	0.15±0.02	0.02±0.01
		5xFAD (n=8)	0.42±0.03	0.05±0.01	0.57±0.11*	0.07±0.05	0.15±0.02	0.02±0.01
**Hippocampus**	3 months	WT (n=8)	0.47±0.05	0.09±0.02	0.58±0.04	0.03±0.01	0.15±0.02	0.03±0.01
		5xFAD (n=8)	0.49±0.03	0.09±0.02	0.59±0.04	0.03±0.00	0.16±0.02	0.02±0.01
	6 months	WT (n=8)	0.41±0.03	0.06±0.01	0.53±0.05	0.06±0.01	0.14±0.02	0.02±0.01
		5xFAD (n=7)	0.40±0.04	0.06±0.01	0.55±0.05	0.06±0.00	0.13±0.02	0.02±0.01
	12 months	WT (n=8)	0.38±0.06	0.06±0.01	0.51±0.03	0.04±0.01	0.14±0.04	0.01±0.01
		5xFAD (n=8)	0.44±0.04	0.07±0.01	0.57±0.06	0.05±0.01	0.17±0.01	0.02±0.01

Urethane (1.5 g/kg) anesthetized mice were infused with [2-^{13}C]acetate for 15 min. The concentrations of ^{13}C-labeled metabolites were measured in tissue extracts in the ^{13}C-edited spectrum using [2-^{13}C]glycine as a reference. Values are presented as Mean±SD. Statistical significance was measured using Multiple unpaired t-tests with the Holm-Sidak method of multiple comparisons. *$p_{adj}<0.05$ when 5xFAD was compared with WT mice. Abbreviations: Asp_{C3}, [3-^{13}C]aspartate; $GABA_{C2}$, [2-^{13}C]GABA; $GABA_{C4}$, [4-^{13}C]GABA; Glu_{C3}, [3-^{13}C]glutamate; Glu_{C4}, [4-^{13}C]glutamate; Gln_{C4}, [4-^{13}C]glutamine.

Figure S1 Amyloid species positively correlate with glial cell activation in 5xFAD mice.

(A-D) Linear regression plots showing a significant positive correlation between soluble Aβ oligomers and GFAP+ astrocyte in the cerebral cortex and hippocampus of 6-month (n=4; purple) and 12-month-old (n=4; orange) 5xFAD mice. **(E-H)** Correlation between soluble Aβ oligomers and IBA1+ microglia in the cortex and hippocampus. **(I-L)** Linear regression between Aβ plaque load and glial cell counts in the cerebral cortex and hippocampus of 6-month-old (purple) and 12-month-old (orange) 5xFAD mice. Each dot represents measurement from an individual animal. Spearman's correlation coefficient (r) and significance values (p) are indicated.

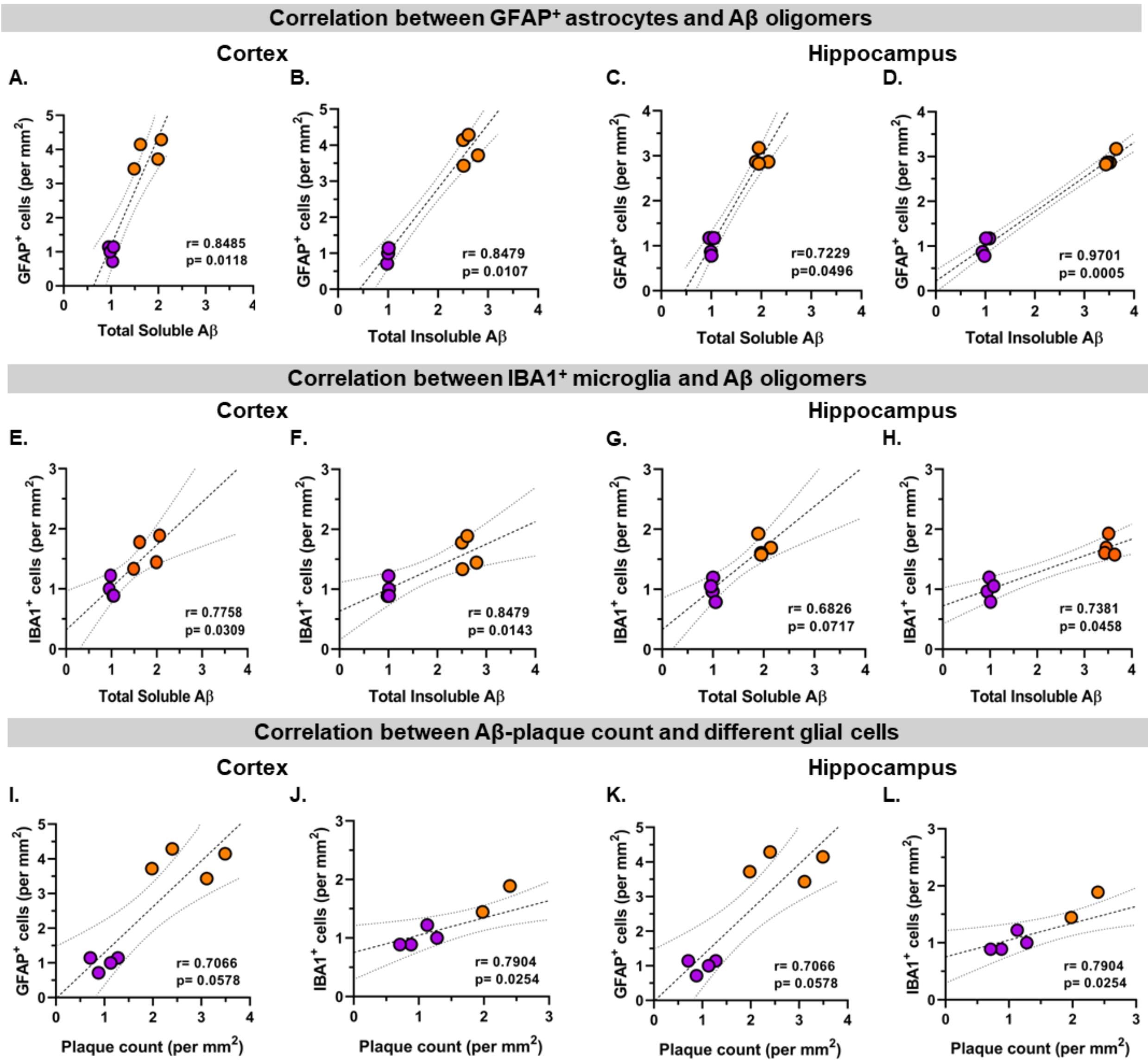

Figure S2 Sensitivity of metabolic impairment and gliosis to amyloid pathology in 5xFAD mice.

(A, B) Heatmaps showing the sensitivity to impairment of neuronal glucose oxidation rates and memory to different amyloid species, including Tris-soluble, GuHCl-soluble, and total $A\beta_{40}$, $A\beta_{42}$, and Aβ plaque burden, and $A\beta_{42}/A\beta_{40}$ ratio in 5xFAD mice. Each cell represents the slope of the linear relationship between the respective measures and different amyloid components. To facilitate comparisons across different amyloid species, average values of individual amyloid species and the $A\beta_{42}/A\beta_{40}$ ratio were normalized to their respective 3-month values and used to estimate their impact on neurometabolism and cognitive function. **(C, D)** Heatmaps showing the sensitivity of astroglial (GFAP+) and microglial (IBA1+) burden to different amyloid biomarkers during disease progression. For sensitivity of gliosis, amyloid biomarker levels and glial cell counts at 12 months were normalized to their respective 6-month values. Numerical values within each cell represent the corresponding slope estimates.

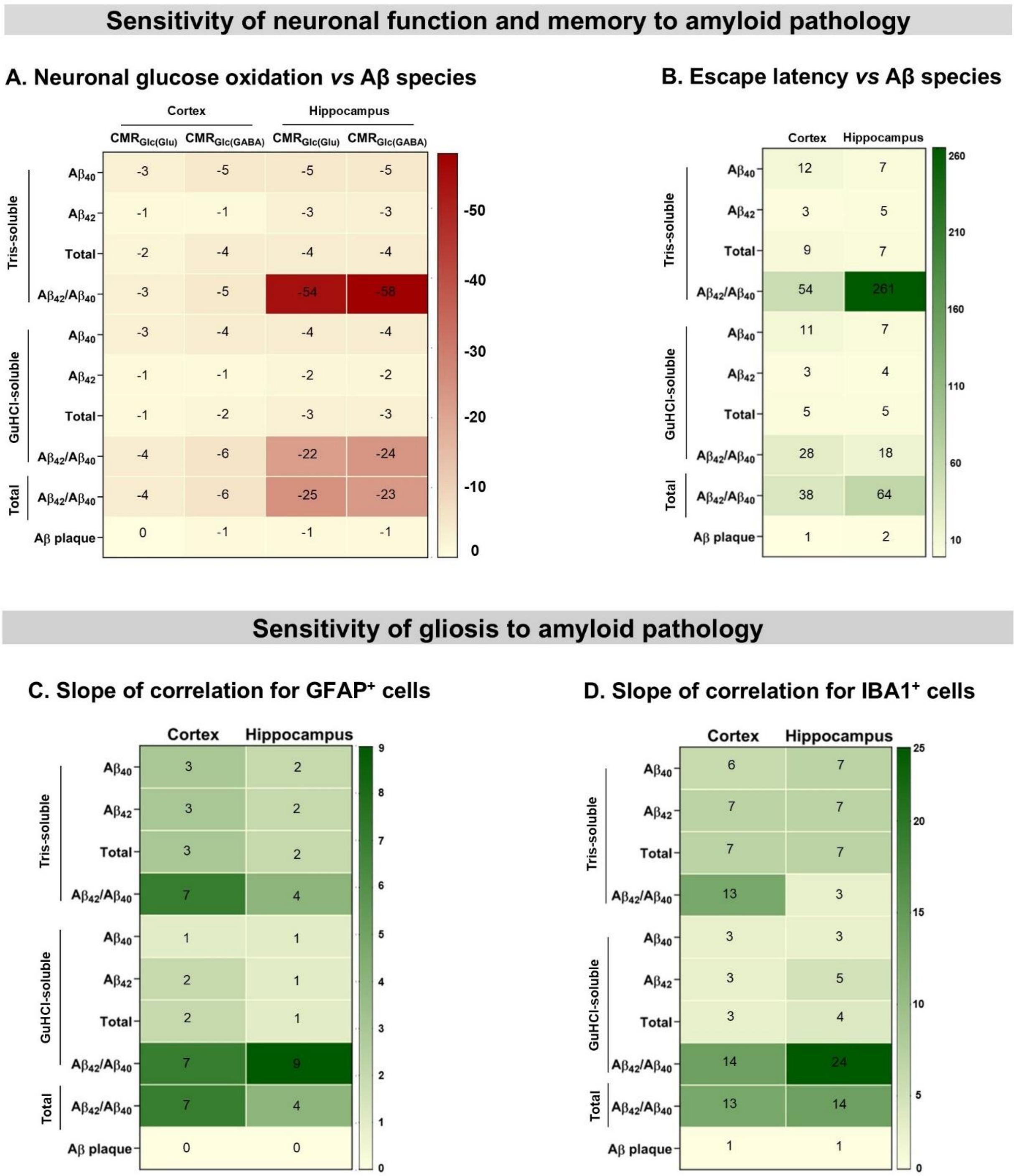

Sensitivity of neuronal function and memory to amyloid pathology
A. Neuronal glucose oxidation vs Aβ species
Cortex
Hippocampus
CMRGlc(Glu) CMRGlc(GABA) CMRGlc(Glu) CMRGlc(GABA)
Tris-soluble
Aβ40 -3 -5 -5 -5
Aβ42 -1 -1 -3 -3
Total -2 -4 -4 -4
Aβ42/Aβ40 -3 -5 -54 -58
GuHCl-soluble
Aβ40 -3 -4 -4 -4
Aβ42 -1 -1 -2 -2
Total -1 -2 -3 -3
Aβ42/Aβ40 -4 -6 -22 -24
Total
Aβ42/Aβ40 -4 -6 -25 -23
Aβ plaque 0 -1 -1 -1
-50 -40 -30 -20 -10 0
B. Escape latency vs Aβ species
Cortex Hippocampus
Tris-soluble
Aβ40 12 7
Aβ42 3 5
Total 9 7
Aβ42/Aβ40 54 261
GuHCl-soluble
Aβ40 11 7
Aβ42 3 4
Total 5 5
Aβ42/Aβ40 28 18
Total
Aβ42/Aβ40 38 64
Aβ plaque 1 2
260 210 160 110 60 10
Sensitivity of gliosis to amyloid pathology
C. Slope of correlation for GFAP+ cells
Cortex Hippocampus
Tris-soluble
Aβ40 3 2
Aβ42 3 2
Total 3 2
Aβ42/Aβ40 7 4
GuHCl-soluble
Aβ40 1 1
Aβ42 2 1
Total 2 1
Aβ42/Aβ40 7 9
Total
Aβ42/Aβ40 7 4
Aβ plaque 0 0
9 8 7 6 5 4 3 2 1 0
D. Slope of correlation for IBA1+ cells
Cortex Hippocampus
Tris-soluble
Aβ40 6 7
Aβ42 7 7
Total 7 7
Aβ42/Aβ40 13 3
GuHCl-soluble
Aβ40 3 3
Aβ42 3 5
Total 3 4
Aβ42/Aβ40 14 24
Total
Aβ42/Aβ40 13 14
Aβ plaque 1 1
25 20 15 10 5 0

Figure S3 Metabolic dysfunction correlates with spatial memory impairment in 5xFAD mice.

(A-F) Correlation between escape latency in the Morris water maze and cerebral metabolic rate of glucose oxidation ($CMR_{Glc(Ox)}$) in the cerebral cortex and hippocampus at different ages in 5xFAD (n=7-8; brown) and WT (n=7-8; grey) mice. **(G, H)** Correlation between escape latency and cerebral metabolic rate of acetate oxidation ($CMR_{Ace(Ox)}$) in the cerebral cortex and hippocampus of 12-month-old 5xFAD (n=8; brown) and WT (n=8; grey) mice. Each dot represents measurement from an individual animal. Pearson's correlation coefficient (r) and significance values (p) are indicated.

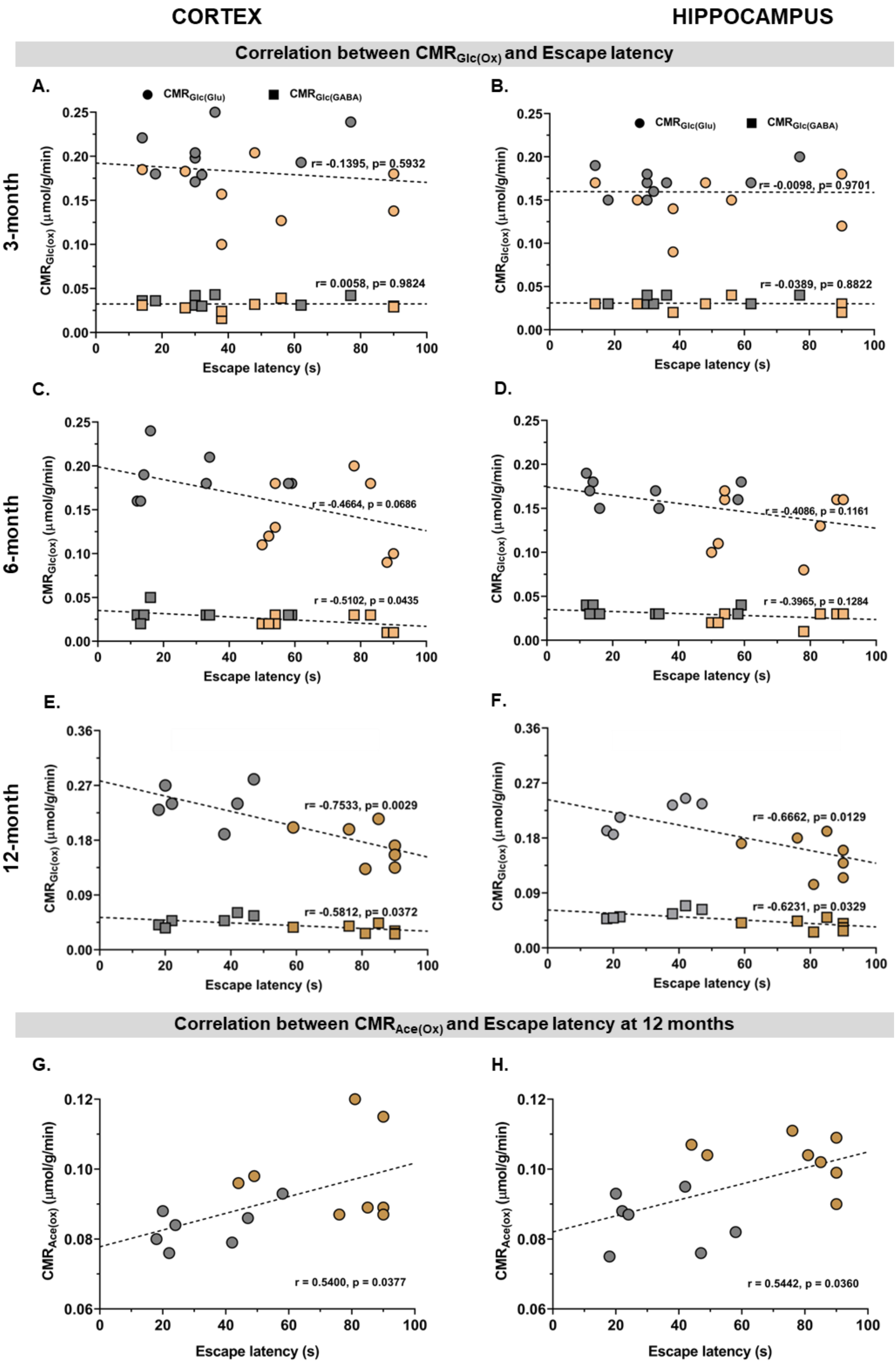
CORTEX
HIPPOCAMPUS
Correlation between CMRGlc(Ox) and Escape latency
A.
B.
C.
D.
E.
F.
3-month
6-month
12-month
CMRGlc(Glu)
CMRGlc(GABA)
CMRGlc(ox) (μmol/g/min)
Escape latency (s)
r= -0.1395, p= 0.5932
r= 0.0058, p= 0.9824
r= -0.0098, p= 0.9701
r= -0.0389, p= 0.8822
r = -0.4664, p = 0.0686
r = -0.5102, p = 0.0435
r = -0.4086, p = 0.1161
r = -0.3965, p = 0.1284
r= -0.7533, p= 0.0029
r= -0.5812, p= 0.0372
r= -0.6662, p= 0.0129
r= -0.6231, p= 0.0329
Correlation between CMRAce(Ox) and Escape latency at 12 months
G.
H.
CMRAce(ox) (μmol/g/min)
Escape latency (s)
r = 0.5400, p = 0.0377
r = 0.5442, p = 0.0360

Figure S4. Immunostaining using anti-GFAP antibody in 3-month-old 5xFAD and WT mice. (A) Representative confocal microscopy images (63×) showing GFAP (green) stained astrocytes in the cerebral cortex and hippocampus. Nuclei were stained with DAPI (blue). **(B, C)** $GFAP^+$ area (%) and astrocyte count (per mm^2) in 3-month-old 5xFAD (n=4) and WT (n=4) mice. Each dot represents the average of measurements from 3 to 4 sections per animal. Data are presented as mean±SD, and group differences were assessed using the Mann-Whitney U-test. Scale bar: 25 μm.

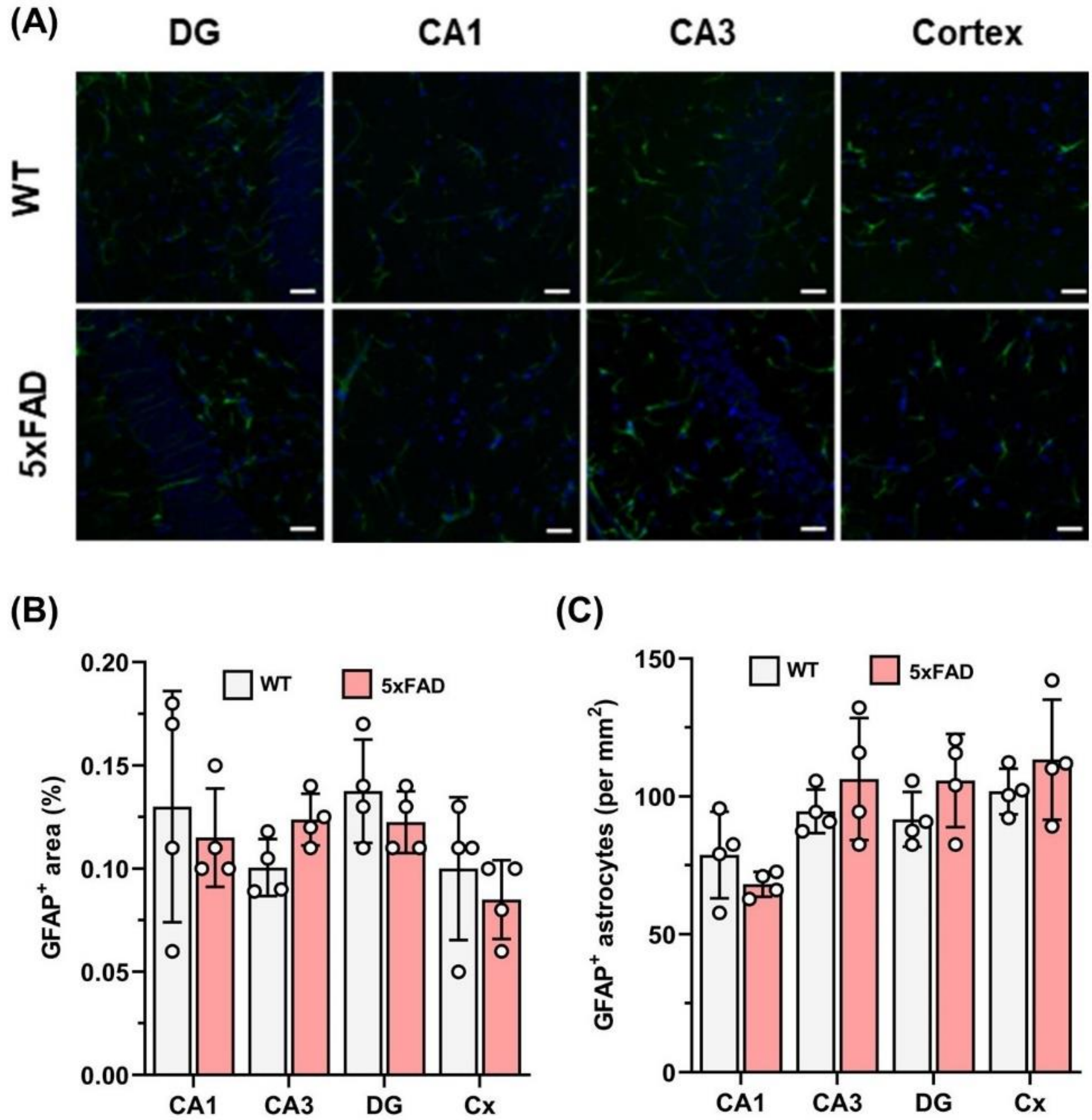

Supplement: Supplementary file 1 [file 13195_2026_2130_MOESM1_ESM.pdf]
